# A CD4-lineage erythro–myeloid progenitor within the adult bone marrow defines an alternative pathway for macrophage generation

**DOI:** 10.1038/s41590-026-02686-1

**Published:** 2026-10-26

**Authors:** Shweta Tikoo, Rohit Jain, Brendon Martinez, Renhua Song, Matthias Wielscher, Carlos Márquez-Burgoa, Anastasiya Hladik, Simone Rizzetto, Lisa E. Shaw, Minoo Ghannadan, Stephanie A. Gerhofer, Andrew J. Mitchell, Maria Elizabeth Torres-Pacheco, Fabio Luciani, Matthias Farlik, Justin J. L. Wong, Sylvia Knapp, Philipp Starkl, Stuart T. Fraser, Steffen Jung, Wolfgang Weninger

**Affiliations:** 1 Department of Dermatology, Medical University of Vienna, Vienna, Austria; 2 Centenary Institute, Faculty of Medicine and Health, The University of Sydney, Sydney, New South Wales Australia; 3 Sydney Medical School, The University of Sydney, Sydney, Australia; 4 Research Division of Infection Biology, Department of Medicine I, Medical University of Vienna, Vienna, Austria; 5 School of Biomedical Sciences, University of New South Wales, Sydney, New South Wales Australia; 6 Department of Chemical and Biomolecular Engineering, Materials Characterization and Fabrication Platform, University of Melbourne, Melbourne, Victoria Australia; 7 School of Medical Sciences, University of Sydney, Sydney, New South Wales Australia; 8 University of Sydney Nano Institute, University of Sydney, Sydney, New South Wales Australia; 9 Department of Immunology and Regenerative Biology, Weizmann Institute of Science, Rehovot, Israel

**Keywords:** Innate immunity, Haematopoiesis

## Abstract

Tissue-resident macrophages originate from distinct developmental sources, but their adult replenishment is thought to depend exclusively on circulating monocytes. Here we identify a previously unrecognized CD4-lineage erythro–myeloid progenitor (EMPROR) in the adult bone marrow that provides an alternative pathway for macrophage generation. EMPROR cells are phenotypically and transcriptionally distinct from hematopoietic stem cells, common myeloid progenitors and monocyte precursors, lacking *Csf1r* and *Ccr2* while expressing *Cd4* and *Kit*. Single-cell RNA sequencing, RNA velocity analysis and lineage assays reveal macrophage, neutrophil, megakaryocytic and erythroid potential. EMPROR cells generate perivascular and tumor-associated macrophages independently of CCR2-mediated monocyte recruitment and contribute to erythroid and granulocytic lineages under inflammatory and neoplastic conditions. Transcriptomic comparisons position EMPROR cells closest to embryonic erythro–myeloid progenitors, indicating the persistence of an embryonic-like program in adult bone marrow. These findings reveal an alternative pathway of adult myelopoiesis that sustains macrophage diversity beyond the classical monocyte lineage.

## Abstract (web summary)

Tissue-resident macrophages originate from distinct developmental sources, but their adult replenishment is thought to depend exclusively on circulating monocytes. Here Tikoo and colleagues identify a CD4-lineage erythro–myeloid progenitor (EMPROR) in the adult bone marrow that provides an alternative pathway for macrophage generation.

## Main

Macrophages represent a heterogeneous family of immune cells involved in regulating tissue homeostasis and inflammatory responses following injury, pathogen invasion and neoplastic transformation^1–4^. It is now widely accepted that macrophages develop through two distinct pathways, depending on their organ of residence and its inflammatory state^2,5–8^. During homeostasis, most tissue-resident macrophages are of prenatal origin and self-renew throughout adulthood independent of bone marrow (BM) input. These prenatally derived macrophages arise from distinct embryonic precursors, the earliest of which are the yolk sac-derived macrophage progenitors (MPs) and erythro–myeloid progenitors (EMPs)^5,6,9–12^. Although MP- and EMP-derived macrophages seed all embryonic organs, the precursors themselves are believed to be consumed during development and have never been identified in adult tissues^5,10^. Postpartum, hematopoietic stem cell (HSC)-derived monocytes replenish several macrophage niches, resulting in ontogenic macrophage heterogeneity within select tissues^9,13,14^. Similarly, in the setting of inflammation or in tumors in adults, circulating monocytes infiltrate tissues, where they differentiate into macrophages^15–17^. Although monocyte-derived macrophages acquire gene expression profiles and features reminiscent of tissue-resident macrophages^18,19^, whether they attain all functional characteristics of tissue-resident macrophages is still unclear^20^. In addition, macrophage populations present within a given microenvironment are not uniform; rather, functionally specialized subpopulations are present depending on their anatomical localization^14,21–24^. During inflammation and cancer, monocytes are widely regarded as the predominant source of newly generated macrophages; however, whether additional precursors contribute to macrophage production has remained elusive^2^.

In this study, we present evidence for the unexpected presence of an EMP-like progenitor in the adult BM. Under certain inflammatory and neoplastic conditions, these cells serve as a rich source of newly formed macrophages, neutrophils and erythrocytes. Moreover, they appear to be the predominant source of distinct macrophage subsets, most prominently perivascular macrophages (PVMs), during inflammatory conditions. Together, these findings suggest the existence of a monocyte-independent pathway for generating macrophages in the adult.

## Results

### BM progenitors generate PVMs during inflammation

We have previously described a distinct population of tissue-resident macrophages termed PVMs, which localize to the perivascular space of postcapillary venules in the dermis^25^. As part of the perivascular extravasation unit, PVMs mediate the recruitment of leukocytes into the skin during inflammation^26^. The precise ontogeny of PVMs remains controversial.

**Fig. 1:**
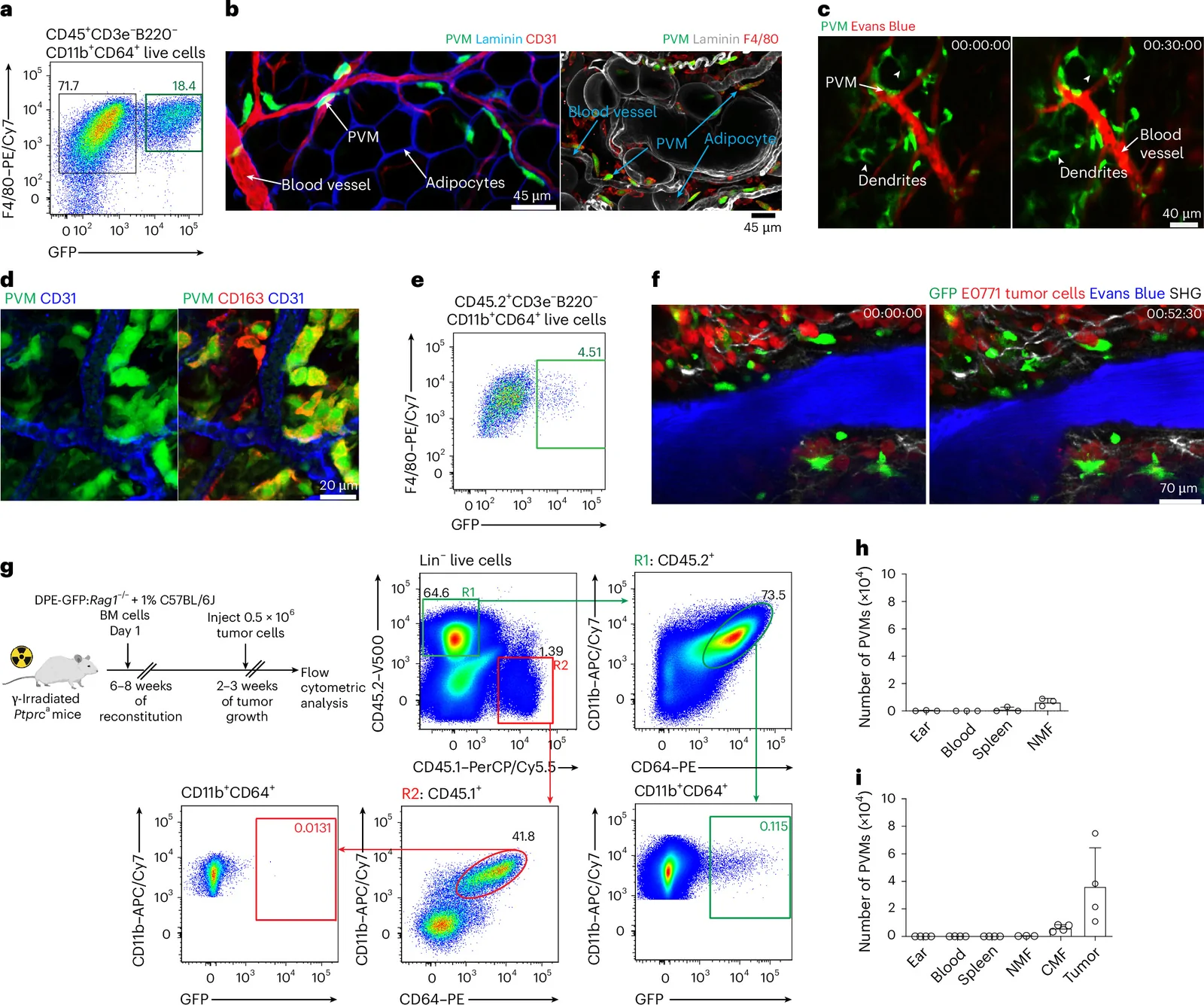
Identification and origin of PVMs. **a**, Flow cytometric identification of CD45^+^CD3e^−^B220^−^CD11b^+^CD64^+^F4/80^+^GFP^−^ and CD45^+^CD3e^−^B220^−^CD11b^+^CD64^+^F4/80^+^GFP^+^ macrophages in naive mammary fat pads of DPE-GFP mice. Data representative of three independent experiments are shown. **b**, Confocal imaging of immunostained mammary fat pad identifying GFP^+^ PVMs. Co-staining with F4/80 is depicted on the right. Data are representative of *n* = 3 mice from three independent experiments. **c**, Intravital imaging of sessile GFP^+^ PVMs within the naive mammary fat pad displaying actively scanning dendrites. Data represent images from three independent experiments. **d**, Confocal imaging of immunostained mammary tumors showing GFP^+^ PVMs along neoangiogenic tumor vasculature. Data represent images from three independent experiments. **e**, Flow cytometric identification of CD45.2^+^CD3e^−^B220^−^CD11b^+^CD64^+^F4/80^+^GFP^−^ and CD45.2^+^CD3e^−^B220^−^CD11b^+^CD64^+^F4/80^+^GFP^+^ macrophages within mammary tumors. A representative flow plot from one of three independent experiments is shown. **f**, Intravital imaging of E0771 mammary tumors transplanted into DPE-GFP:*Rag1*^−/−^ mice demonstrates the presence of GFP^+^ PVMs around the tumor vasculature. Data are representative of intravital studies with *n* = 3 mice from three independent experiments. SHG, second harmonic generation. **g**, Flow cytometric analysis of tumors from DPE-GFP:*Rag1*^−/−^ → *Ptprc*^a^ chimeric mice identifying the presence of donor-derived GFP^+^ and GFP^−^ macrophages; *n* = 4 mice. Data are representative of one of two independent experiments. Lineage markers consist of CD90 and B220. CD45.1^−^ and CD45.2^−^ cells represent tumor cells. **h**, Quantification of donor-derived PVMs in various tissues under homeostasis, 7–8 months after BM reconstitution; *n* = 3 mice. Data are representative of two independent experiments; NMF, naive mammary fat pads. Bars represent mean ± s.d. **i**, Enumeration of donor-derived PVMs in various tissues after tumor implantation, 10 weeks after BM reconstitution; *n* = 4 mice for all tissues except naive mammary fat pad (*n* = 3 mice); CMF, contralateral mammary fat pad of tumor-bearing mice. Bars represent mean ± s.d. Data are representative of one of two independent experiments. Schematic in **g** created in BioRender; Jain, R. https://biorender.com/9z2f7np (2026). Source data

**Extended Data Fig. 1:**
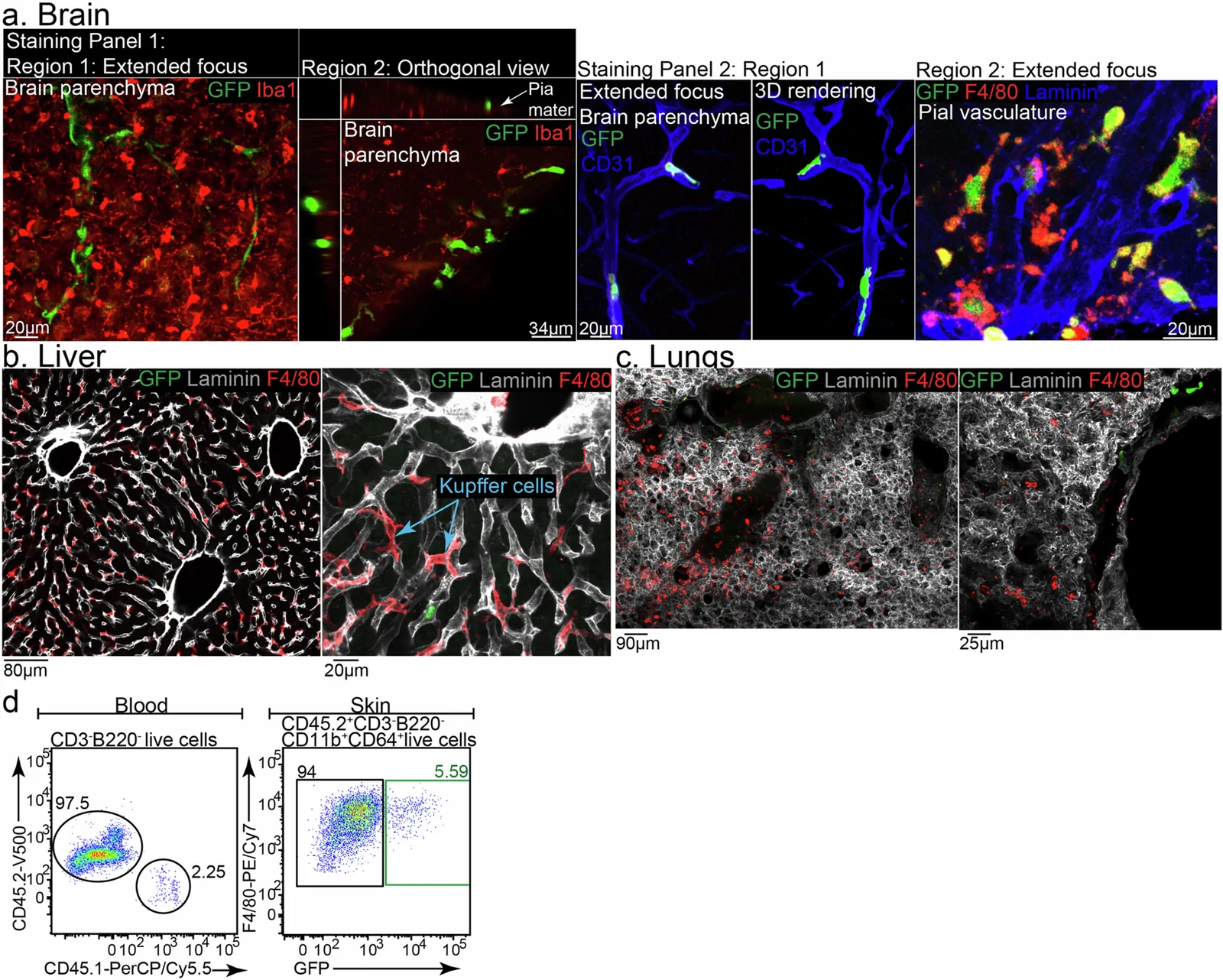
Distribution of GFP^+^ perivascular macrophages in various murine tissues. **a**, Confocal imaging of immunostained brain sections from DPE-GFP:*Rag1*^−/−^ mice showing the absence of GFP expression in microglial cells (marked by Iba1 expression) and distinct expression of GFP in perivascular macrophages along the pial vasculature; **b**, Confocal imaging of immunostained liver sections from DPE-GFP:*Rag1*^−/−^ mice reveals the absence of GFP expression in Kupffer cells; **c**, Confocal imaging of immunostained lung sections from DPE-GFP:*Rag1*^−/−^ mice highlighting the absence of GFP expression in lung macrophages; **d**, Flow cytometric analysis of the ear skin from DPE-GFP:*Rag1*^−/−^→ *Ptprc*^*a*^ chimeras demonstrating partial replacement of tissue macrophages by CD45.2^+^CD3e^−^B220^−^CD11b^+^CD64^+^F4/80^+^GFP^+^ macrophages over 15 months, n = 3; Panels a, b and c, imaging data is representative of tissue harvested from n = 3 mice from two independent experiments.

The selective identification of PVMs is greatly facilitated in the transgenic mouse strain DPE-GFP^27^, in which regulatory elements of the *Cd4* locus drive the expression of green fluorescent protein (GFP^25^; Supplementary Fig. 1a). Besides T cells and plasmacytoid dendritic cells (pDCs), PVMs, but not interstitial macrophages, selectively express GFP in these mice. In addition to the dermis^25^, CD11b^+^CD64^+^F4/80^+^GFP^+^ PVMs are present in other organs, including the mammary fat pad, the meninges and the superficial layer of central nervous system (CNS) cortex, in DPE-GFP and DPE-GFP:*Rag1*^−/−^ mice (Fig. 1a and Extended Data Fig. 1a). Similar to the skin, confocal microscopy of inguinal mammary fat pads revealed that GFP^+^ cells lined the blood vessels (Fig. 1b, left). Co-staining with anti-F4/80 confirmed the identity of these cells to be macrophages (Fig. 1b, right). Intravital multiphoton microscopy illustrated that these cells were sessile with dendrites that actively probed the surrounding tissue microenvironment (Fig. 1c and Supplementary Video 1). In contrast to PVMs, other tissue-resident macrophages, such as microglia, Kupffer cells and lung macrophages, did not express GFP in DPE-GFP:*Rag1*^−^^/^^−^ mice (Extended Data Fig. 1a–c).

In pathological settings, such as the tumor milieu, increased numbers of PVMs can be found at sites of neoangiogenesis^28^. To investigate the origin of PVMs under such conditions, we used a mouse breast cancer model. Following implantation of syngeneic E0771 medullary breast adenocarcinoma cells^29,30^ into the inguinal mammary fat pad of DPE-GFP:*Rag1*^−/−^ mice, we observed numerous GFP^+^CD163^+^ macrophages within tumors, particularly in areas of pronounced vascular development at the tumor periphery (Fig. 1d). Phenotypically, these GFP^+^ macrophages were CD45^+^CD3e^−^B220^−^CD11b^+^CD64^+^F4/80^+^ (Fig. 1e). Intravital multiphoton microscopy confirmed the presence of GFP^+^ PVMs in close proximity to the tumor vasculature (Fig. 1f and Supplementary Video 2).

Based on the fact that PVMs and potentially their precursors can be conveniently tracked in DPE-GFP mice, we sought to determine their turnover kinetics in both normal and diseased tissues. Initially, we performed BM chimera studies in which irradiated, congenic *Ptprc*^a^ (CD45.1) mice received BM from DPE-GFP:*Rag1*^−/−^ mice (CD45.2; note that these mice lack GFP^+^ T cells), mixed with 1% BM from wild-type C57BL/6J mice (Fig. 1g, left). This was done to reconstitute the adaptive immune system (T and B cells) while restricting GFP expression to PVMs and their potential progenitors. Seven to 8 months after reconstitution, very few, if any, GFP^+^ cells were present in the ear skin, circulation, spleen or mammary fat pads, consistent with the idea that PVMs are long-lived and/or self-renew (Fig. 1h). Nevertheless, after 15 months, approximately 5% of PVMs in the skin were GFP^+^, revealing slow replenishment, presumably from the BM (Extended Data Fig. 1d). When E0771 breast cancer cells were implanted into DPE-GFP:*Rag1*^−/−^ → *Ptprc*^a^ BM chimeras (reconstituted for 6–8 weeks), GFP^+^ macrophages developed within the tumors, but not in other organs (Fig. 1g,i). These data show that one or more PVM precursors exist in the adult BM.

**Extended Data Fig. 2:**
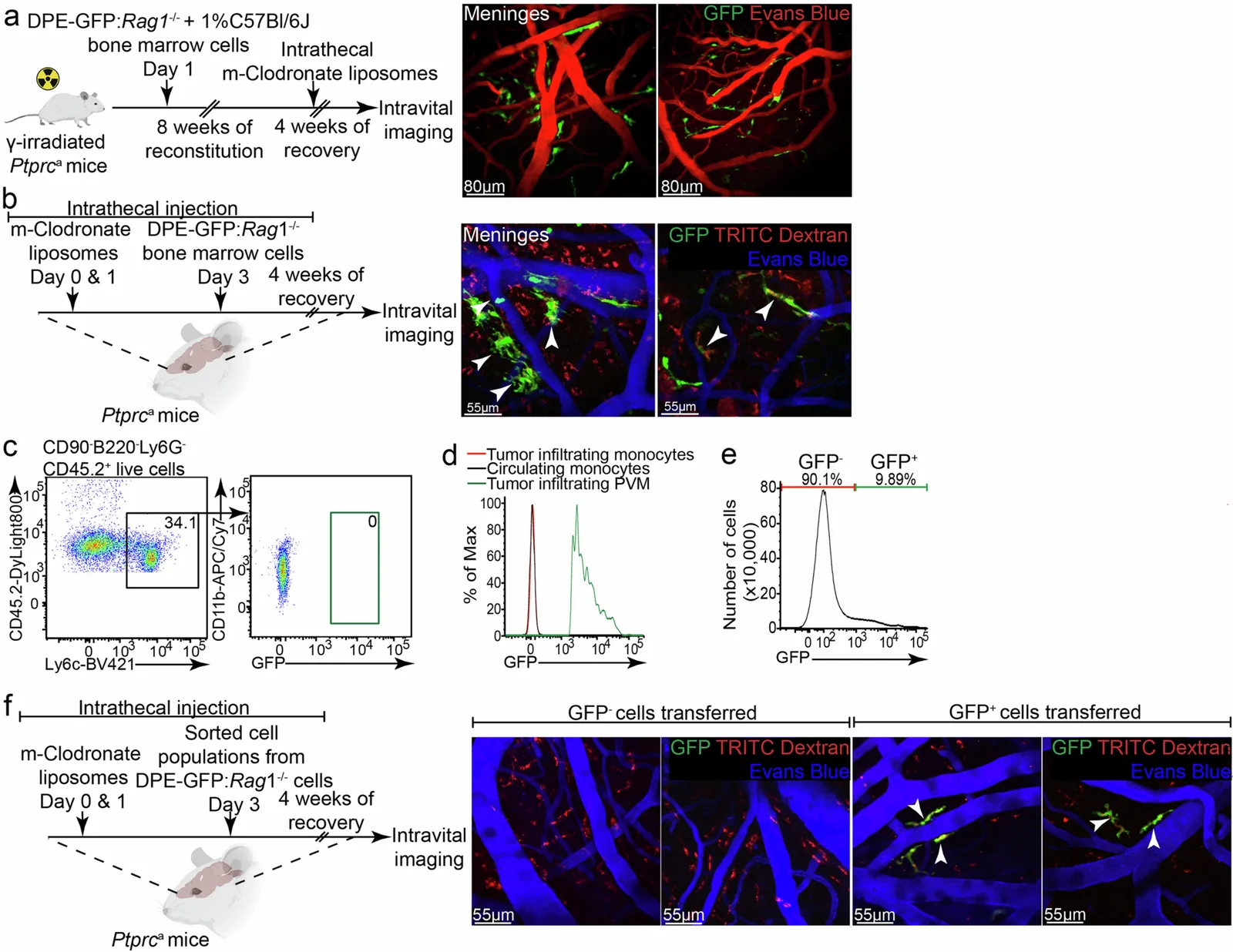
Origin of GFP^+^ macrophages post-inflammation. **a**, Intravital imaging of DPE-GFP: *Rag1*^−/−^→ *Ptprc*^*a*^ chimeric mice 4 weeks post-depletion of macrophages by mannosylated clodronate liposomes identifies the presence of numerous donor-derived GFP^+^ perivascular macrophages in the CNS. Image data is representative of n = 4 mice from four independent chimera experiments; **b**, Intravital imaging of brains of mice injected with unfractionated DPE-GFP:*Rag1*^−/−^ bone marrow cells post-meningeal macrophage depletion identifies the presence of several GFP^+^TRITC dextran^+^ PVMs. This experiment establishes the intrathecal precursor transfer methodology used in subsequent experiments. Image data is representative of n = 3 mice from two independent experiments; **c**, Flow cytometric analysis of blood from DPE-GFP:*Rag1*^−/−^→*Ptprc*^*a*^ chimeric mice, highlighting the absence of GFP in donor-derived monocytes. n = 4 mice. Data is representative of one of two independent experiments; **d**, Histogram plot delineating the expression of GFP in circulating and tumor-infiltrating monocytes; tumor-infiltrating GFP^+^ PVM were used as an internal positive control for GFP expression; n = 4 mice. The data is representative of one of two independent experiments. **e**, Histogram plot displaying the presence of GFP^+^ and GFP^−^cells within the DPE-GFP bone marrow. Histogram plot is representative of several independent experiments (n > 5). **f**, Intravital imaging of brains of mice injected with sorted GFP^−^ and GFP^+^ bone marrow cells from DPE-GFP:*Rag1*^−/−^ mice. GFP^+^ PVMs were only detected in mice injected with GFP^+^ bone marrow cells. Image data is representative of three independent experiments wherein the CNS was repleted with GFP^+^ (n = 4) and GFP^−^ (n = 3) sorted bone marrow cells from DPE-GFP:*Rag1*^−/−^ mice. Schematics in **a**, **b** and **f** created in BioRender; Jain, R. https://biorender.com/9z2f7np (2026).

To validate the BM origin of PVMs, we next developed a depletion model in which intrathecal (i.t.) administration of mannosylated clodronate liposomes led to the depletion of meningeal macrophages, thereby creating a niche for various precursors to seed and develop. Four weeks after i.t. clodronate liposome injection into DPE-GFP:*Rag1*^−/−^ → *Ptprc*^a^ BM chimeras, intravital multiphoton microscopy in the CNS revealed the presence of GFP^+^ PVMs lining the postcapillary venules within the meningeal space (Extended Data Fig. 2a). This indicates that donor BM precursors have the capacity to replenish the PVM pool in the CNS. We corroborated this finding by direct i.t. transfer of bulk BM cells from DPE-GFP × *Rag1*^−/−^ mice into *Ptprc*^a^ mice that were previously depleted of meningeal macrophages (Extended Data Fig. 2b). After allowing 4 weeks of reconstitution, intravital microscopy revealed the presence of GFP^+^ PVMs lining the postcapillary venules within the meningeal space (Extended Data Fig. 2b). Together, these data indicate that BM-derived precursors are a potential source of PVMs in the setting of tumorigenesis or macrophage depletion in adult animals.

### An EMP in adult BM

As PVMs arose from the adult BM, the most likely PVM precursor candidate would be circulating Ly6C^hi^CD11b^+^ monocytes. However, GFP-expressing monocytes were never observed in DPE-GFP:*Rag1*^−/−^ → *Ptprc*^a^ BM chimeras (Extended Data Fig. 2c,d). While this does not exclude monocytes, as they may turn on GFP expression during their differentiation into macrophages, it is also conceivable that PVMs may be derived from an alternative cell source. Indeed, flow cytometric analysis of BM of DPE-GFP:*Rag1*^−/−^ mice revealed the presence of GFP^+^ cells (Extended Data Fig. 2e). Moreover, when DPE-GFP:*Rag1*^−/−^ BM was fractionated into GFP^+^ and GFP^−^ cells and transferred i.t. into meningeal macrophage-depleted mice, only the GFP^+^ fraction gave rise to a network of GFP^+^ macrophages lining blood vessels (Extended Data Fig. 2f), morphologically reminiscent of PVMs in DPE-GFP mice. GFP^+^ cells readily accumulated i.t. administered tetramethylrhodamine isothiocyanate (TRITC)-dextran (Extended Data Fig. 2f), indicating that these macrophages are functionally mature.

**Fig. 2:**
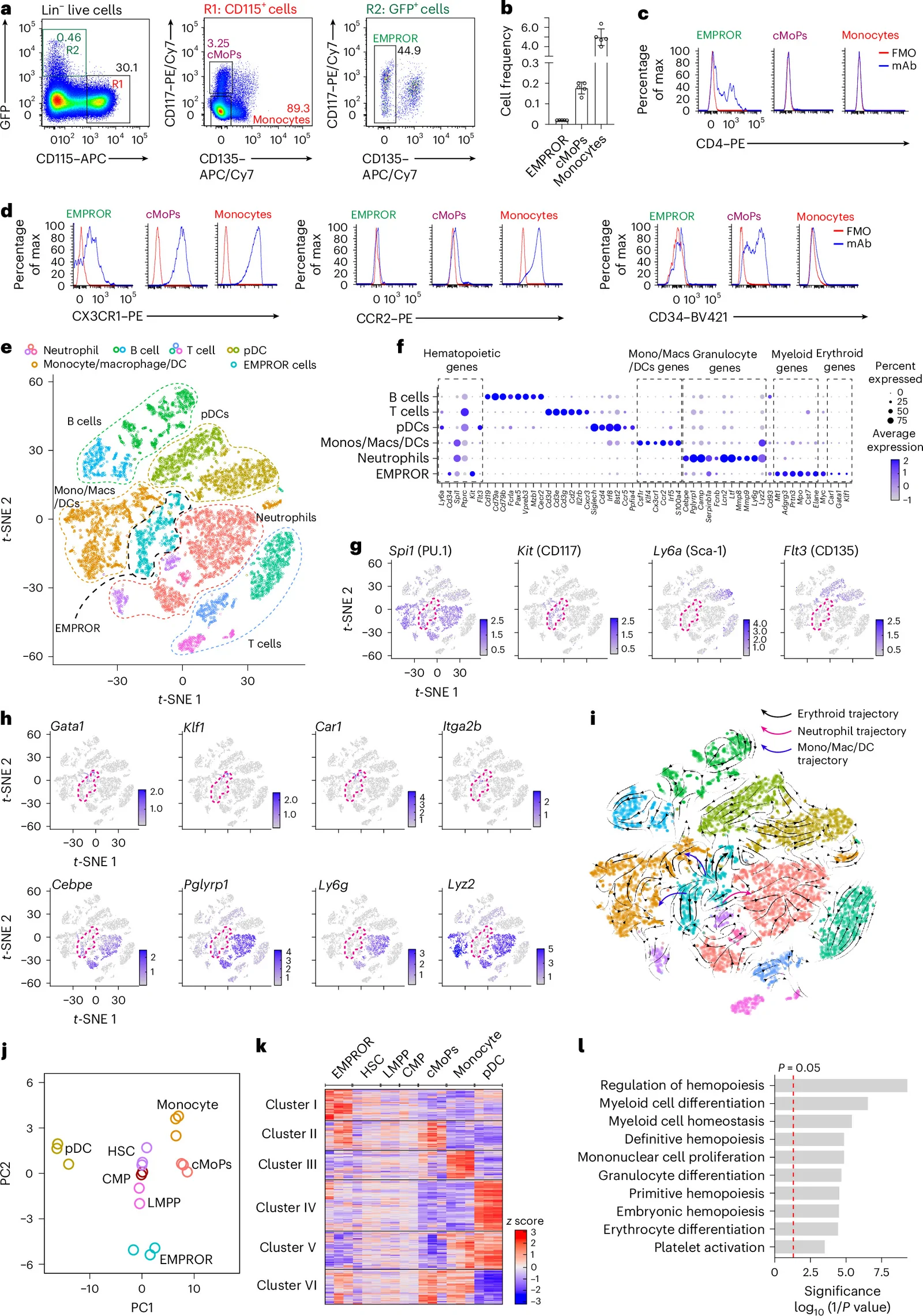
Identification and characterization of EMPROR cells. **a**, Flow cytometric analysis of DPE-GFP:*Rag1*^−/−^ BM highlighting the presence of cMoPs, monocytes and EMPROR cells. Lineage markers comprise CD3e, Ly6G, NK1.1, CD19 and Siglec-H. Data are representative of five independent experiments. **b**, Cell frequency of cMoPs, monocytes and EMPROR cells as a percentage of total live cells in adult BM. Data are cumulative of five independent experiments. Bars represent mean ± s.d. **c**,**d**, Surface expression of CD4 (**c**) and CCR2, CX3CR1 and CD34 (**d**) on cMoPs, monocytes and EMPROR cells. Data are representative of three independent experiments. **e**, *t*-SNE plot of hematopoietic cells from DPE-GFP BM showing a distinct EMPROR cluster. The plot was generated by assimilating scRNA-seq data from sorted CD45^+^(with BM cells; see Methods for details), GFP^+^ and EMPROR cells sorted from DPE-GFP BM. **f**, Bubble plots showing expression of hematopoietic and developmental genes across cell clusters. **g**, *t*-SNE plots showing expression of myeloid marker *Spi1* but not the stem cell marker *Sca1* within the EMPROR cluster. EMPROR cells express varying levels of *Kit* (CD117) but do not express *Flt3* (CD135). **h**, *t*-SNE plots highlighting the expression of several erythrocytic and thrombocytic markers in the EMPROR cluster. The cluster also shows expression of neutrophilic markers, such as the transcription factor *Cebpe* and *Pglyrp1*, but is devoid of mature markers such as *Ly6g* and *Lyz2*. **i**, RNA trajectory analysis of the hematopoietic clusters defined in **e**, highlighting EMPROR trajectories toward erythroid, neutrophil and monocyte (Mono)/macrophage (Mac)/DC clusters. **j**, Two-dimensional PCA of HSCs (light violet), LMPPs (magenta), CMPs (dark red), cMoPs (light red), monocytes (orange), pDCs (dark yellow) and EMPROR cells (cyan). cMoP, monocyte, pDC and EMPROR cell data were generated from three independent sorts (*n* = 5–7 mice pooled per sort). Publicly available datasets were used for HSCs, LMPPs and CMPs. ComBat^59,60^, based on the empirical Bayes method, was applied to the values normalized by variance-stabilizing transformation from DESeq2 for the batch effect adjustment between samples^59,60^. **k**, *k*-Means clustering analysis of differentially expressed genes (*P* < 0.05 after Benjamini–Hochberg correction, fold change of >2) in HSCs, LMPPs, CMPs, cMoPs, monocytes, pDCs and EMPROR cells identified six unique clusters, with cluster I being specific for EMPROR cells. Expression values for each differentially expressed gene are normalized across all samples by the *z*-score transformation method. **l**, Gene Ontology analysis of cluster I from **k** showing enrichment of functions associated with erythroid and myeloid hematopoiesis; FMO, fluorescence minus one; mAb, monoclonal antibody; PC, principal component. Source data

**Extended Data Fig. 3:**
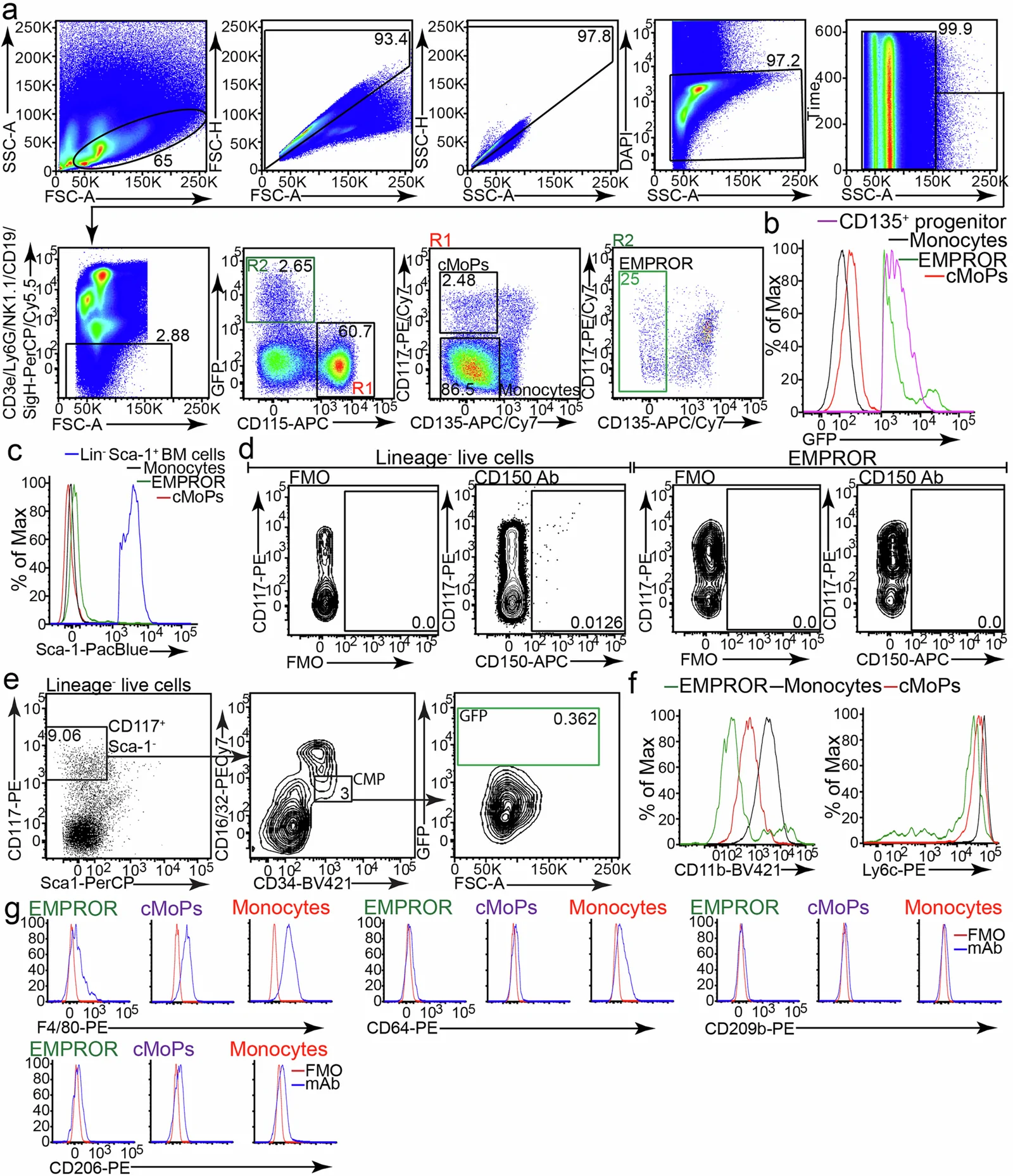
Characterization of EMPROR cells. **a**, Flow cytometric analysis of bone marrow from DPE-GFP mice identifies EMPROR cells as SSC^lo^DAPI^−^CD3e^−^Ly6G^−^NK1.1^−^CD19^−^Siglec-H^−^CD115^−^CD135^−^GFP^+^CD117^+^ cells. Representative flow plots from one of several independent experiments (n > 5) are shown; **b**, Histogram plots depicting the expression of GFP in cMoPs, monocytes, CD135 precursor, and EMPROR cells (n > 5); **c**, Histogram plots showing the surface expression of Sca-1 on cMoPs, monocytes, and EMPROR. Bone marrow cells not expressing lineage markers but exhibiting surface expression of Sca-1 have been overlaid as an internal positive control. Lineage markers consist of CD3e, Ly6G, NK1.1, CD19, and Siglec-H; **d**, Dot plots representing the lack of surface expression of CD150 on EMPROR cells. Bone marrow cells not expressing lineage markers but exhibiting surface expression of CD150 have been displayed as an internal positive control. Lineage markers consist of CD3e, Ly6G, NK1.1, CD19, and Siglec-H; **e**, Flow cytometric analysis of DPE-GFP bone marrow reveals the absence of GFP expression in common myeloid progenitors (CMP)^36^. Lineage markers consist of CD3e, CD4, CD8, B220, IgM, Ter119, CD127, CD19, and Gr-1 (ref. ^36^); **f**, Histogram plots demonstrating the expression of CD11b and Ly6C by cMoPs, monocytes and EMPROR within the adult bone marrow; **g**, Histogram plots displaying the expression of F4/80, CD64, CD206 and CD209b on cMoPs, monocytes and EMPROR cells. Representative flow plots from one of three independent experiments are shown in panel **c** - **g**. Note that for panels **a**, **f**, and **g**, the lineage cocktail comprised CD3e, Ly6G, NK1.1, CD19, and Siglec-H antibodies.

Flow cytometric analysis of the BM of DPE-GFP and DPE-GFP:*Rag1*^−/−^ mice showed that Lin^−^CD115(CSF1R)^+^CD117(cKit)^−^CD135(FLT3)^−^ monocytes and their precursors, including Lin^−^CD115^+^CD117^+^CD135^+^ monocyte DC precursors^31,32^ and Lin^−^CD115^+^CD117^+^CD135^−^ common monocyte precursors (cMoPs)^33^, did not express GFP (Fig. 2a). Rather, GFP expression within the lineage-negative (CD3e^−^Ly6G^−^NK1.1^−^CD19^−^Siglec-H^−^) fraction was restricted to a small but distinct population of CD115^−^CD117^+^CD135^−^ cells (Fig. 2a and Extended Data Fig. 3a,b). A second population of Lin^−^GFP^+^ cells, which expressed CD135, represented lymphocyte progenitors (Fig. 2a and Extended Data Fig. 3a,b); these latter cells were not considered further in the present work. For simplicity, we refer to Lin^−^GFP^+^CD115^−^CD117^+^CD135^−^ cells henceforth as EMPROR (for Erythro–Myeloid PRogenitOR) cells (Fig. 2a and Extended Data Fig. 3a), based on the evidence that they display phenotypic and functional properties previously ascribed to embryonic EMP cells (detailed below). Lack of Sca-1 and CD150 expression^34,35^ distinguishes EMPROR cells from HSCs (Extended Data Fig. 3c,d). Additional analysis revealed that bona fide common myeloid progenitors (CMPs)^36^ did not express GFP in DPE-GFP mice (Extended Data Fig. 3e). EMPROR cells were found to be numerically rare compared to cMoPs and monocytes (Fig. 2b). A proportion of EMPROR cells was positive for CD4, CD11b and Ly6C (Fig. 2c and Extended Data Fig. 3f) but negative for CCR2 and displayed low/intermediate CX3CR1 expression (Fig. 2d). The low CD34 expression further distinguished EMPROR cells from CMPs (Fig. 2d). Moreover, EMPROR cells did not express mature macrophage markers, including F4/80, CD64, CD206 and CD209b (Extended Data Fig. 3g).

### Transcriptional profiling of EMPROR cells

**Extended Data Fig. 4:**
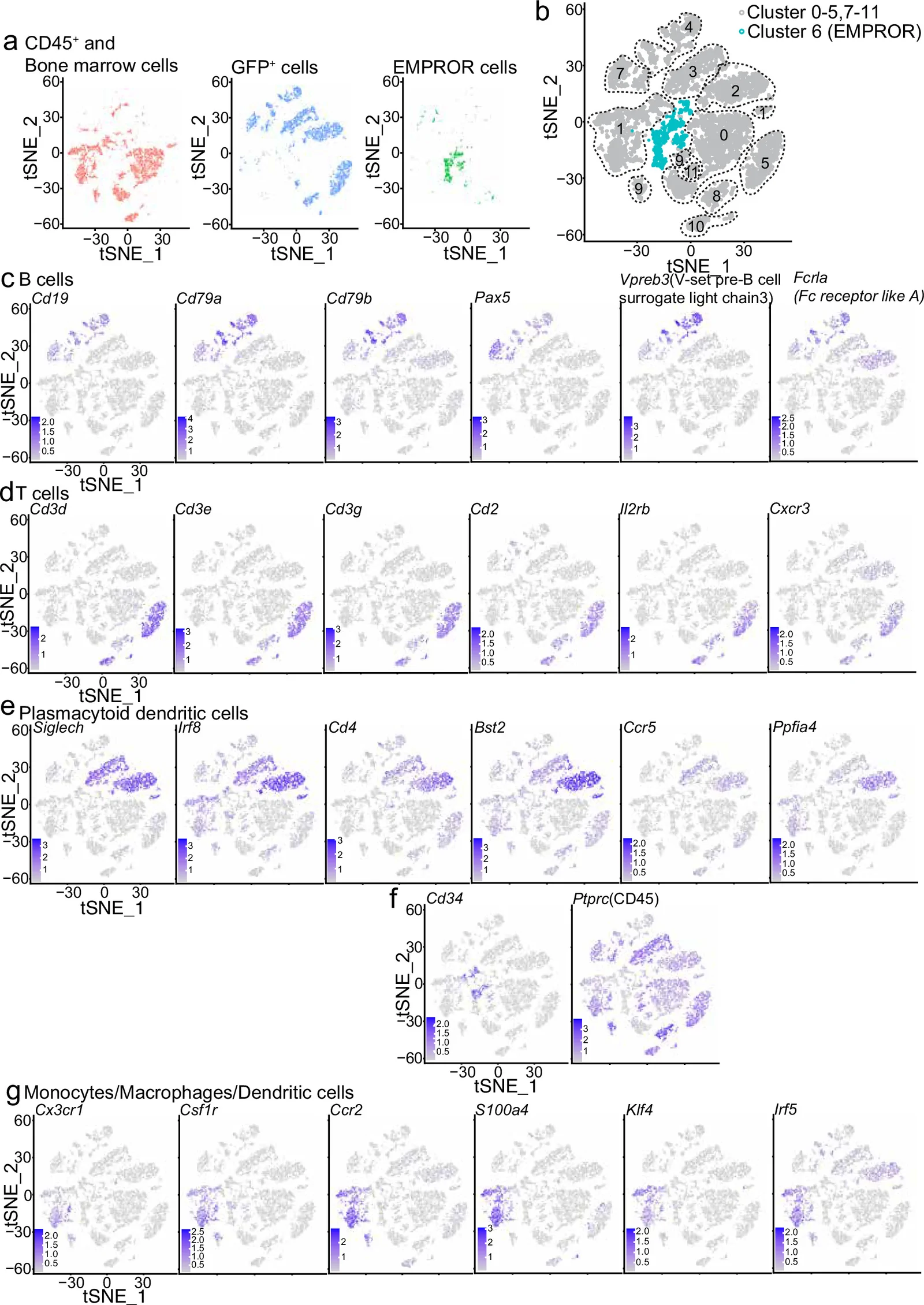
*t*-SNE analysis of EMPROR cells. **a**, *t*-SNE plots depicting the distribution of sorted CD45^+^ and bone marrow cells (CD45^+^ + BM), GFP^+^ cells, and EMPROR cells from each sample; **b**, *t*-SNE plot highlighting the presence of EMPROR cluster 6 (cyan) present in the dataset generated by assimilating single-cell RNA Seq data from sorted CD45^+^ + BM, GFP^+^, and EMPROR cells sorted from DPE-GFP bone marrow; **c**-**e** *t*-SNE plots confirming the expression of specific markers within B cells, T cells, and pDC single-cell clusters. The expression of these and other markers was used to assign lineages in Fig. 2e, f; **f**, *t*-SNE plots highlighting the expression of commonly known hematopoietic genes, namely *Cd34* and *Ptprc* (CD45), in various clusters; **g**, *t*-SNE plots confirming the expression of specific monocyte, macrophage, and dendritic cell markers. Panels **c**-**g**, common names, names of protein derivatives, or CD classification for the genes have been provided in parentheses.

To obtain a more complete understanding of the nature of EMPROR cells, we performed single-cell RNA sequencing (scRNA-seq). The transcriptional profile of EMPROR cells was compared to sorted pan-GFP-expressing cells and CD45^+^ cells spiked with whole BM cells (CD45^+^ + BM) from DPE-GFP mice (see details in Methods). As expected, *t*-distributed stochastic neighbor embedding (*t*-SNE) analysis identified several distinct cell clusters present within the CD45^+^ + BM and GFP^+^ samples (Extended Data Fig. 4a). By contrast, a predominant cluster was observed for EMPROR cells (Extended Data Fig. 4a). In the combined dataset, 12 clusters were present, with cluster VI representing EMPROR cells (Fig. 2e and Extended Data Fig. 4b).

The EMPROR cluster lacked expression of common transcriptional markers for B cells (Fig. 2f and Extended Data Fig. 4c), T cells (Fig. 2f and Extended Data Fig. 4d) and pDCs (Fig. 2f and Extended Data Fig. 4e). While EMPROR cells expressed transcripts for PU.1 (*Spi1*), *Cd34* and CD117 (*Kit*), they lacked expression of Sca-1 (*Ly6a*), CD135 (*Flt3*) and CD45 (*Ptprc*) transcripts (Fig. 2f,g and Extended Data Fig. 4f). Additionally, EMPROR cells lacked expression of *Cx3cr1*, *Csf1r*, *Ccr2*, *S100a4*, *Klf4* and *Irf5* transcripts (Fig. 2f and Extended Data Fig. 4g), thereby marking them as a distinct population compared to the conventional monocyte, macrophage and DC lineages.

**Extended Data Fig. 5:**
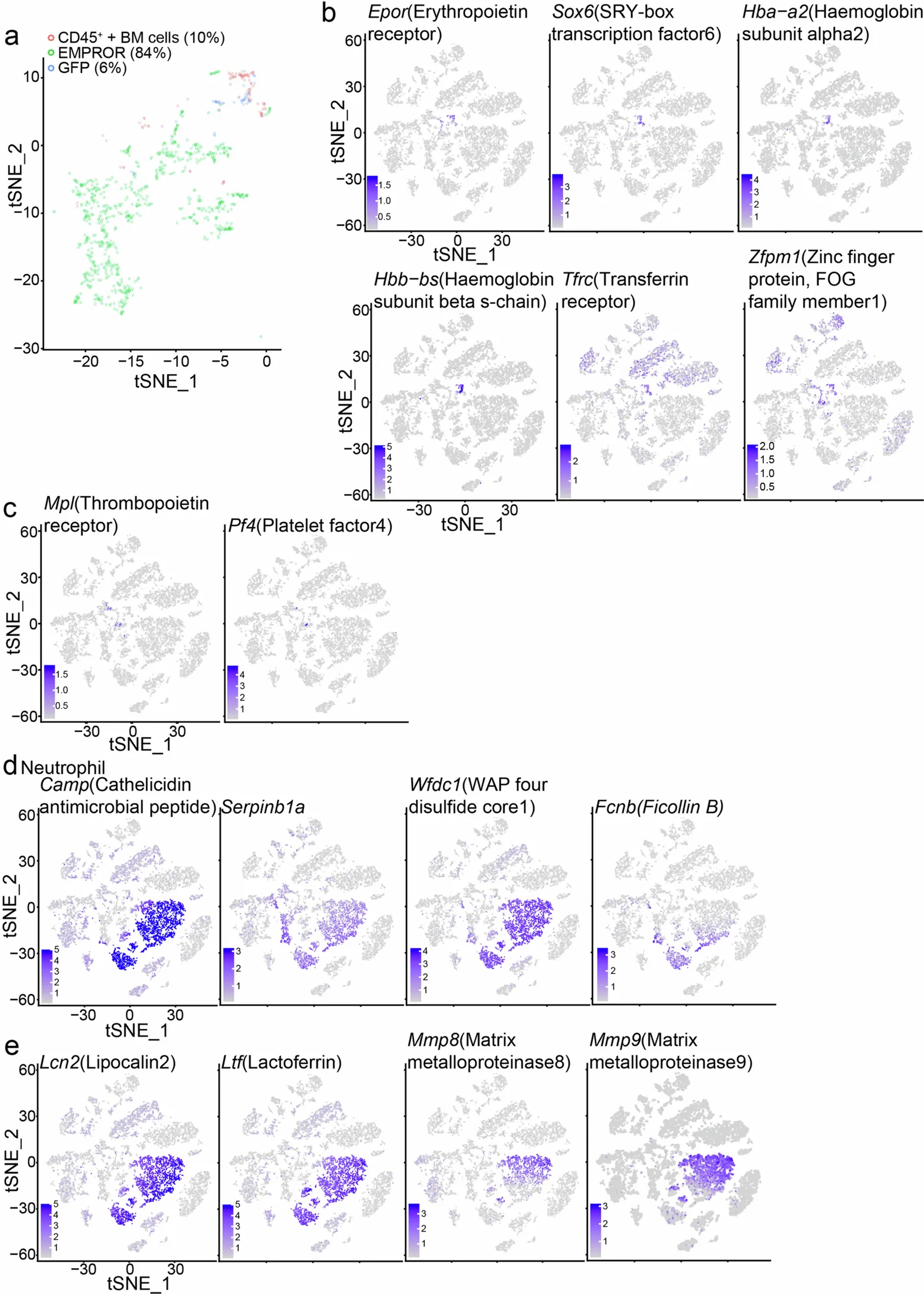
Subset-specific gene expression within single cell clusters. **a**, *t*-SNE plot for EMPROR cluster 6 emphasizing the inclusion of cells from CD45^+^ + BM (10%) and GFP^+^ (6%) samples; **b**, *t*-SNE plots demonstrating the expression of erythroid markers by EMPROR Cluster 6; **c**, *t*-SNE plots confirming the expression of platelet lineage markers within the transcriptome of a subset of EMPROR cells present in Cluster 6; **d**, *t*-SNE plots highlighting the expression of early neutrophil lineage markers by various cells within the EMPROR and neutrophil cluster; **e**, *t*-SNE plots marking the lack of expression of mature neutrophil markers within the EMPROR cluster. Panels **b**-**e**, common names, names of protein derivatives, or CD classification for the genes have been provided in parentheses.

When focusing on the EMPROR cluster, we observed inclusion of a small fraction of cells from the CD45^+^ + BM and GFP^+^ cell samples (Extended Data Fig. 5a). This small fraction of cells was predominantly composed of erythrocytic and megakaryocytic lineages, as revealed by the expression of erythropoietin receptor (*Epor*), SRY-box transcription factor 6 (*Sox6*), hemoglobin subunit-α (*Hba-a2*) and hemoglobin subunit-β (*Hbb-bs*; Extended Data Fig. 5b) along with the expression of transferrin receptor (*Tfrc*) and zinc finger protein FOG family member 1 (*Zfpm1*; Extended Data Fig. 5b). When assessed for erythrocytic and megakaryocytic markers, we observed expression of key transcription factors involved in erythrocytic lineage development, such as GATA binding factor 1 (*Gata1*) and Kruppel-like factor 1 (*Klf1*), as well as carbonic anhydrase 1 (*Car1*), a metalloenzyme critical for hemoglobin function in erythrocytes (Fig. 2h), by a small fraction of the EMPROR cells. Furthermore, a fraction of the cells also expressed the megakaryocytic lineage genes encoding CD41b (*Itga2b*; Fig. 2h), thrombopoietin receptor (*Mpl*) and platelet factor 4 (*Pf4*; Extended Data Fig. 5c). These findings highlight the presence of erythrocytic and megakaryocytic transcripts within a small fraction of EMPROR cells. EMPROR cells also showed expression of certain genes previously described for neutrophil precursors^37^, for example, *Cebpe*, *Pglyrp1*, *Camp*, *Serpinb1a*, WAP four disulfide core 1 (*Wfdc1*) and ficolin B (*Fcnb*; Fig. 2f,h and Extended Data Fig. 5d). Nonetheless, these cells were devoid of other neutrophil markers, such as lipocalin-2 (*Lcn2*), lactoferrin (*Ltf*), matrix metalloproteinase-8 (*Mmp8*), matrix metalloproteinase-9 (*Mmp9*), lymphocyte antigen G6 (*Ly6g*) and lysozyme-M (*Lyz2*; Fig. 2f,h and Extended Data Fig. 5e).

The presence of erythrocytic, megakaryocytic and myeloid transcripts within the EMPROR cell cluster highlighted the multilineage potential for these cells. Therefore, using RNA velocity, we assessed their developmental trajectories in silico. Using scVelo^38^, RNA velocities derived from the dynamic modeling of the dataset highlighted trajectories from EMPROR cells toward erythroid, neutrophil and monocyte/macrophage/DC clusters (Fig. 2i). Together, these findings underscore the unique transcriptional signature of EMPROR cells along with their in silico predicted multilineage potential.

**Extended Data Fig. 6:**
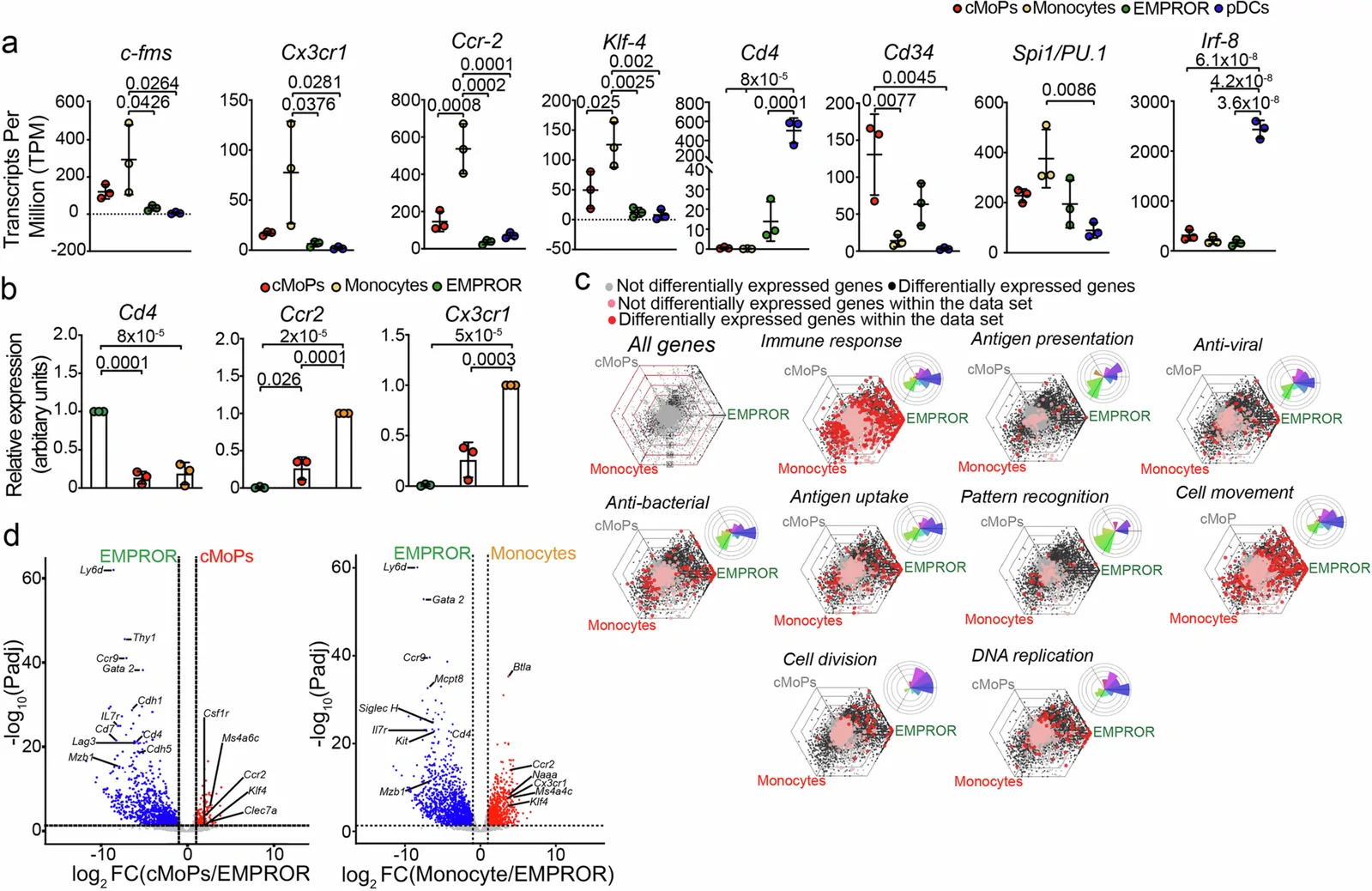
Transcriptomic analysis of EMPROR cells. **a**, Transcriptomic analysis of various genes associated with myeloid cell development and function, emphasizing the unique transcriptional signature of EMPROR cells when compared to cMoPs and monocytes. The data were analyzed from the sequencing of sorted precursors from three independent sort experiments (n = 5-7 mice/sort). One-way ANOVA with Tukey’s multiple comparisons test was used to determine statistical significance with a 95% confidence interval. Bars represent mean ± s.d. **b**, RT-qPCR analysis confirming the differential expression of *Cd4*, *Ccr2*, and *Cx3cr1* mRNA in EMPROR cells compared to cMoPs and monocytes. The data were generated from RT-qPCR of sorted precursors from three independent sort experiments (n = 4-5 mice/sort). One-way ANOVA with Tukey’s multiple comparisons test was used to determine statistical significance with a 95% confidence interval. Bars represent mean ± s.d. **c**, Hexagonal and rose diagrams highlighting the unique gene expression profile of EMPROR cells when compared to cMoPs and monocytes; **d**, Volcano plots depicting select differentially expressed genes between EMPROR cells and cMoPs and monocytes. Source data

To develop a deeper insight into the transcriptional signature of EMPROR cells, we next performed global transcriptomic analyses of EMPROR cells, cMoPs, monocytes and mature pDCs isolated from DPE-GFP:*Rag1*^−/−^ BM. pDCs served as a control owing to their expression of GFP in DPE-GFP mice^39^. Similar to the single-cell analysis, the bulk sequencing data corroborated the low abundance of transcripts for *C-fms* (*Csf1r*), *Cx3cr1*, *Ccr2* and *Klf4* in EMPROR cells compared to cMoPs and monocytes (Extended Data Fig. 6a). We also found higher expression of *Cd4* in EMPROR cells, low expression levels of *Cd34* and intermediate expression of PU.1 (Extended Data Fig. 6a). *Irf8* expression was negligible in all the cell types except pDCs (Extended Data Fig. 6a). Quantitative PCR with reverse transcription (RT–qPCR) validated the differential expression of *Cd4*, *Ccr2* and *Cx3cr1* in cMoPs, monocytes and EMPROR cells (Extended Data Fig. 6b). To compare the global gene profiles of EMPROR cells with cMoPs and monocytes, we used hexagonal and rose diagrams^18^. These analyses revealed the distinct expression profiles of select gene sets in EMPROR cells (Extended Data Fig. 6c). Furthermore, volcano plots highlighted the differential gene expression within the analyzed populations (Extended Data Fig. 6d). For example, pairwise comparison between EMPROR cells and cMoPs highlighted the expression of *Cd4* in EMPROR cells versus high expression of *Csf1r* and *Ccr2* in cMoPs (Extended Data Fig. 6d). Similarly, pairwise comparison between EMPROR cells and monocytes demonstrated the expression of *Kit* in EMPROR cells versus high expression of *Ccr2* and *Cx3cr1* in monocytes (Extended Data Fig. 6d).

The unique transcriptional program of EMPROR cells was highlighted by two-dimensional principal component analysis (PCA) compared to the known macrophage precursors, cMoPs and monocytes (Fig. 2j). Further bioinformatic comparison with transcriptomic datasets from previously described progenitor populations, including HSCs^40^, lymphoid primed multipotent progenitors (LMPPs)^40^ and CMPs^41^, underscored the distinct transcriptional profile of EMPROR cells (Fig. 2j). Unsupervised *k*-means clustering analysis of differentially expressed genes present within EMPROR cells, HSCs, LMPPs, CMPs, cMoPs, monocytes and pDCs identified six unique clusters (Fig. 2k and Supplementary Table 1). While clusters II, V and VI were shared between several populations and clusters III and IV were specific to monocytes and pDCs, respectively, only cluster I was specific for EMPROR cells (Fig. 2k and Supplementary Table 1). Gene Ontology analysis of cluster I identified enrichment of several biological pathways, including those involved in hematopoiesis and myeloid cell and erythrocytic development (Fig. 2l and Supplementary Table 1). Together, these data highlight that EMPROR cells are distinct from known hematopoietic and myeloid precursors based on phenotypic and transcriptional analyses.

### Lineage potential of EMPROR cells

**Fig. 3:**
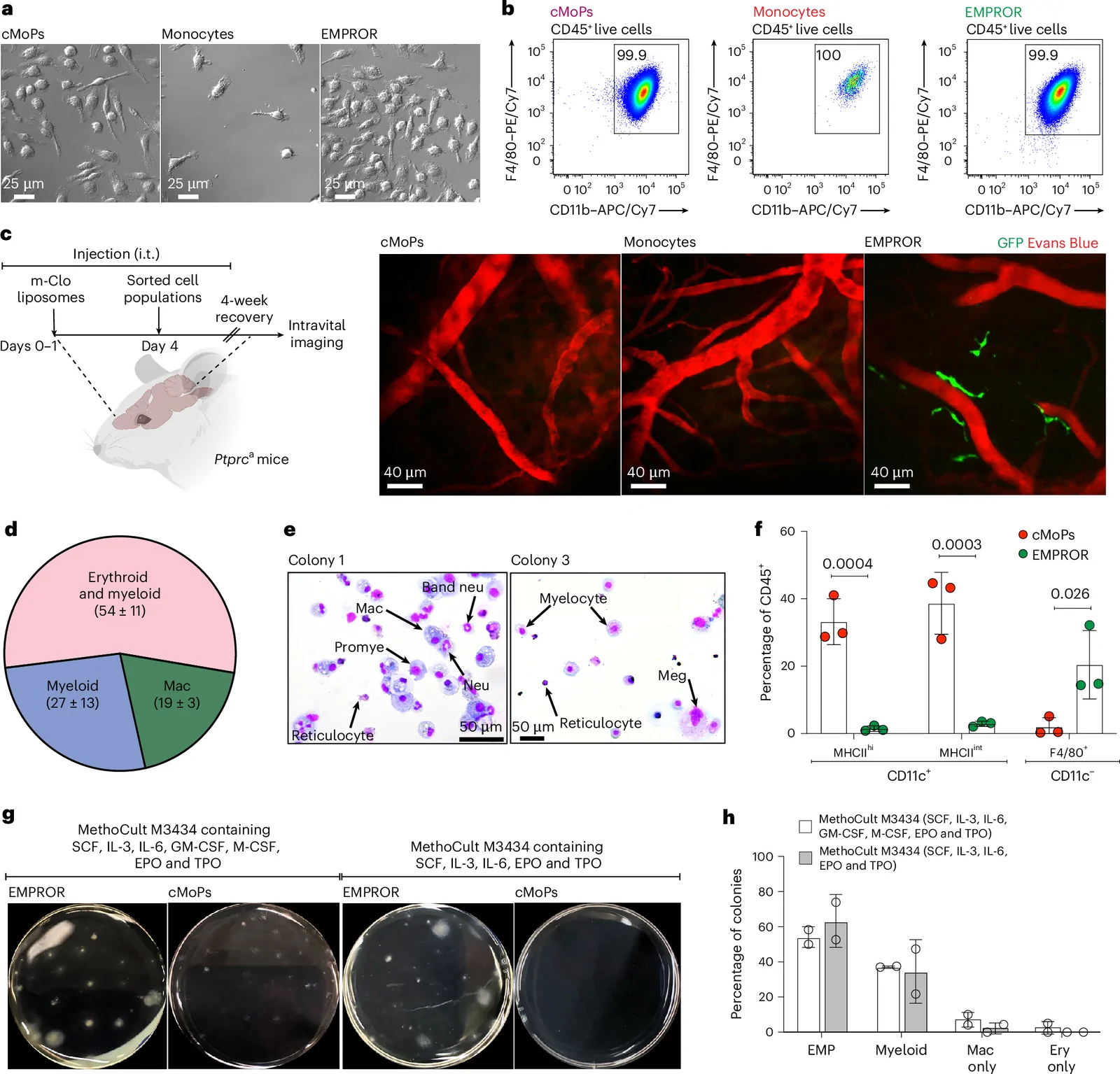
Lineage potential of EMPROR cells. **a**, Confocal differential interference contrast imaging of sorted cMoPs, monocytes and EMPROR cells cultured in complete RPMI medium containing M-CSF. Representative images from one of two independent experiments are shown. **b**, Flow cytometric identification of CD45^+^CD11b^+^F4/80^+^ macrophages following culture. Representative flow plots from one of three independent experiments are shown. **c**, Intravital imaging of meningeal vasculature following i.t. transfer of sorted cMoPs, monocytes and EMPROR cells confirming the presence of GFP^+^ PVMs following EMPROR transfer. Data are representative of three independent experiments wherein the CNS was repleted with EMPROR cells (*n* = 3 mice), cMoPs and monocytes (*n* = 4) sorted from DPE-GFP:*Rag1*^−/−^ BM; m-Clo, mannosylated clodronate. **d**, Pie chart displaying the proportion of macrophage (Mac), myeloid (macrophage and neutrophil) and erythro–myeloid (erythroid with macrophage and neutrophil lineages) colonies generated after culture of sorted EMPROR cells in MethoCult GF M3434 containing transferrin, SCF, IL-3, IL-6 and EPO, supplemented with TPO, GM-CSF and M-CSF. Data are cumulative of three independent sort and cell culture experiments with a total of 34 individual colonies analyzed by flow cytometry. The values represent the percentage of colonies (mean) ± s.d. for each population. **e**, Giemsa staining of colonies illustrating erythroid as well as myeloid lineage potential generated after culture of sorted EMPROR cells (cultured as described in **d**). Images are representative of 2 of 20 separate colonies from four independent experiments. **f**, DC lineage potential of EMPROR cells and cMoPs. Data are cumulative of three independent sort and culture experiments and were analyzed by two-way analysis of variance (ANOVA) with Šídák’s multiple comparisons test to determine statistical significance with a 95% confidence interval. Bars represent mean ± s.d. **g**, Representative macroscopic images of in vitro culture of sorted cMoPs and EMPROR cells in MethoCult GF M3434 containing transferrin, SCF, IL-3, IL-6 and EPO, supplemented with TPO, in the presence or absence of GM-CSF and M-CSF. **h**, Erythro–myeloid potential of EMPROR cells in MethoCult GF M3434 containing transferrin, SCF, IL-3, IL-6 and EPO and supplemented with TPO, in the presence or absence of GM-CSF and M-CSF. Data are cumulative of two independent experiments. Bars represent mean ± s.d*.*; Band neu, band neutrophil; Ery, erythrocyte; Meg, megakaryocyte; Neu, neutrophil; Promye, promyelocyte. Schematic in **c** created in BioRender; Jain, R. https://biorender.com/9z2f7np (2026). Source data

To test the macrophage lineage potential of EMPROR cells in vitro, we sorted them to high purity and cultured them in the respective differentiation medium. cMoPs and monocytes were used as experimental controls. All cultured cell populations generated macrophages, with cMoPs (Fig. 3a,b), and EMPROR cells (Fig. 3a,b and Supplementary Fig. 1b), showing robust expansion of CD45^+^CD11b^+^F4/80^+^ cells (Fig. 3b). The in vivo potential to generate GFP^+^ macrophages was tested in the meningeal macrophage depletion model (Fig. 3c). Flow-sorted cMoPs, monocytes and EMPROR cells were administered i.t. 3–4 days after mannosylated clodronate liposome treatment, followed by imaging 4 weeks later (Fig. 3c). Only EMPROR cells generated GFP^+^ PVMs within the brain (Fig. 3c), demonstrating their unique potential to give rise to this specialized macrophage subset under the tested condition.

**Extended Data Fig. 7:**
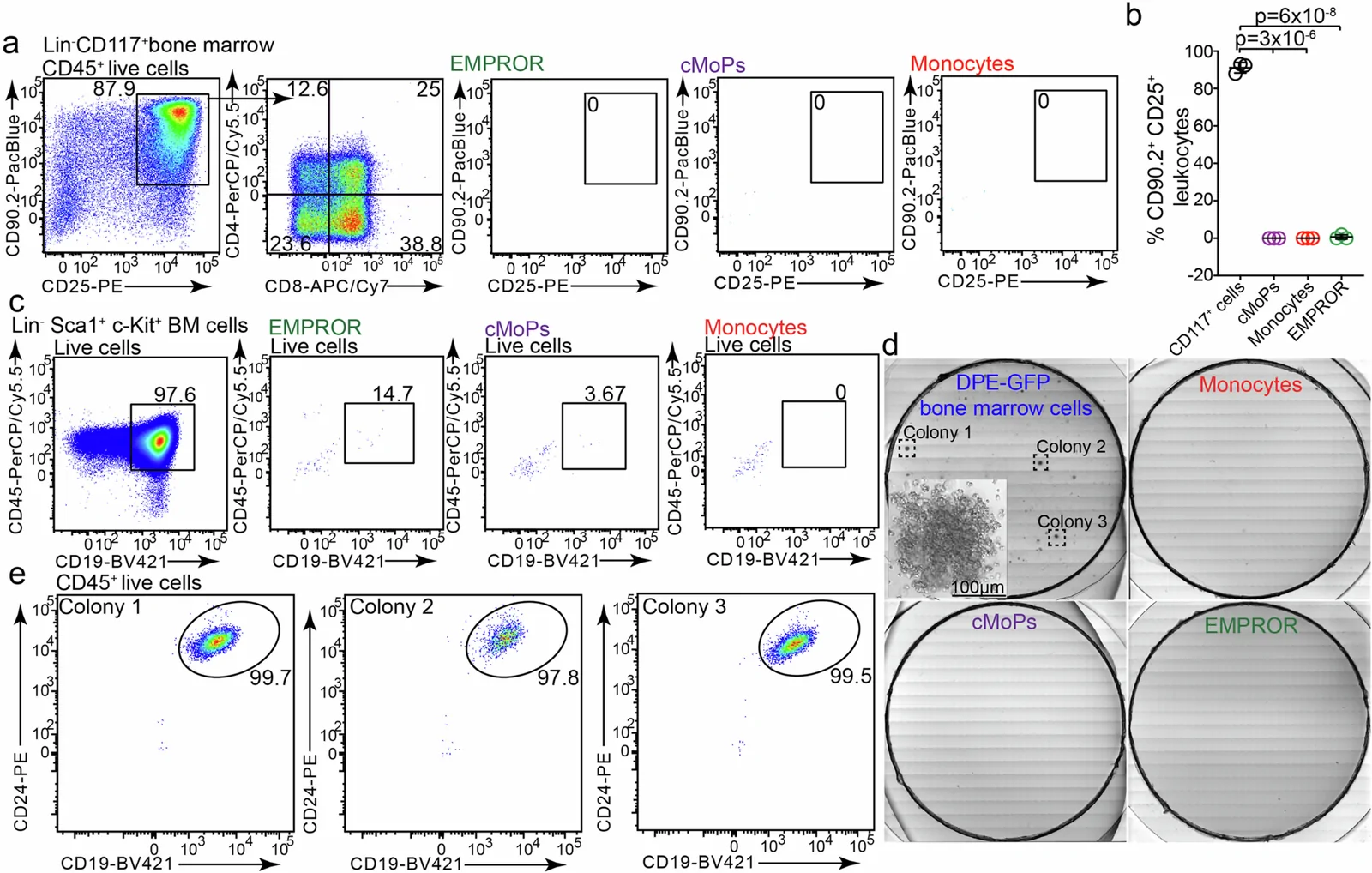
In vitro differentiation potential of adult EMPROR cells. **a**, Flow cytometric analysis of sorted cell populations from the bone marrow of DPE-GFP mice post-culture on OP9 and OP9- DL1 stromal cells corroborates the inability of these precursors to differentiate into T cells. Lin^−^CD117^+^ bone marrow cells were used as a positive control. Representative flow plots from one of three independent experiments, see methods for lineage markers; **b**, Graph representing the lack of T cell potential of sorted cMoPs, monocytes, and EMPROR cells from DPE-GFP bone marrow when cultured employing the OP9 and OP9-DL-1 system. Sorted lin^−^CD117^+^ bone marrow cells were used as a positive control. Data are cumulative of three independent experiments. One-way ANOVA with Tukey’s multiple comparisons test was used to determine statistical significance with a 95% confidence interval. Bars represent mean ± s.d. **c**, Flow cytometric analysis of sorted cell populations from the bone marrow of DPE-GFP mice post-culture on OP9 stromal cells illustrates the absence of B cell lineage commitment. Lin^−^Sca-1^+^c-Kit^+^ bone marrow cells were employed as a positive control; **d**, Confocal imaging of B cell MethoCult™ plates post-culture of sorted cell populations from the bone marrow of DPE-GFP mice. The images highlight the lack of any B cell colonies in plates containing sorted cells; a few colonies were observed in the positive control plate containing unfractionated bone marrow cells. **e**, Flow cytometry of colonies obtained from the unfractionated bone marrow sample, positive control (Panel d) highlights the expression of B cell markers (CD24^+^ CD19^+^). Representative flow plot of 3 colonies from one of three independent experiments. Source data

To assess their ability to give rise to T cells, EMPROR cells, cMoPs, monocytes as well as Lin^−^CD117^+^ BM cells (positive control) were cultured on OP9 and OP9-DL1 stromal cells as described previously^42^. Other than Lin^−^CD117^+^ BM cells, all cell populations failed to generate T cells (Extended Data Fig. 7a,b). To decipher their B cell differentiation potential, we used the OP9 coculture methodology as well as B cell MethoCult^42,43^ (Extended Data Fig. 7c–e). None of the tested cell populations, except the positive control, generated B cells (Extended Data Fig. 7c–e). These observations suggest that EMPROR cells lack an appreciable lymphoid potential.

**Extended Data Fig. 8:**
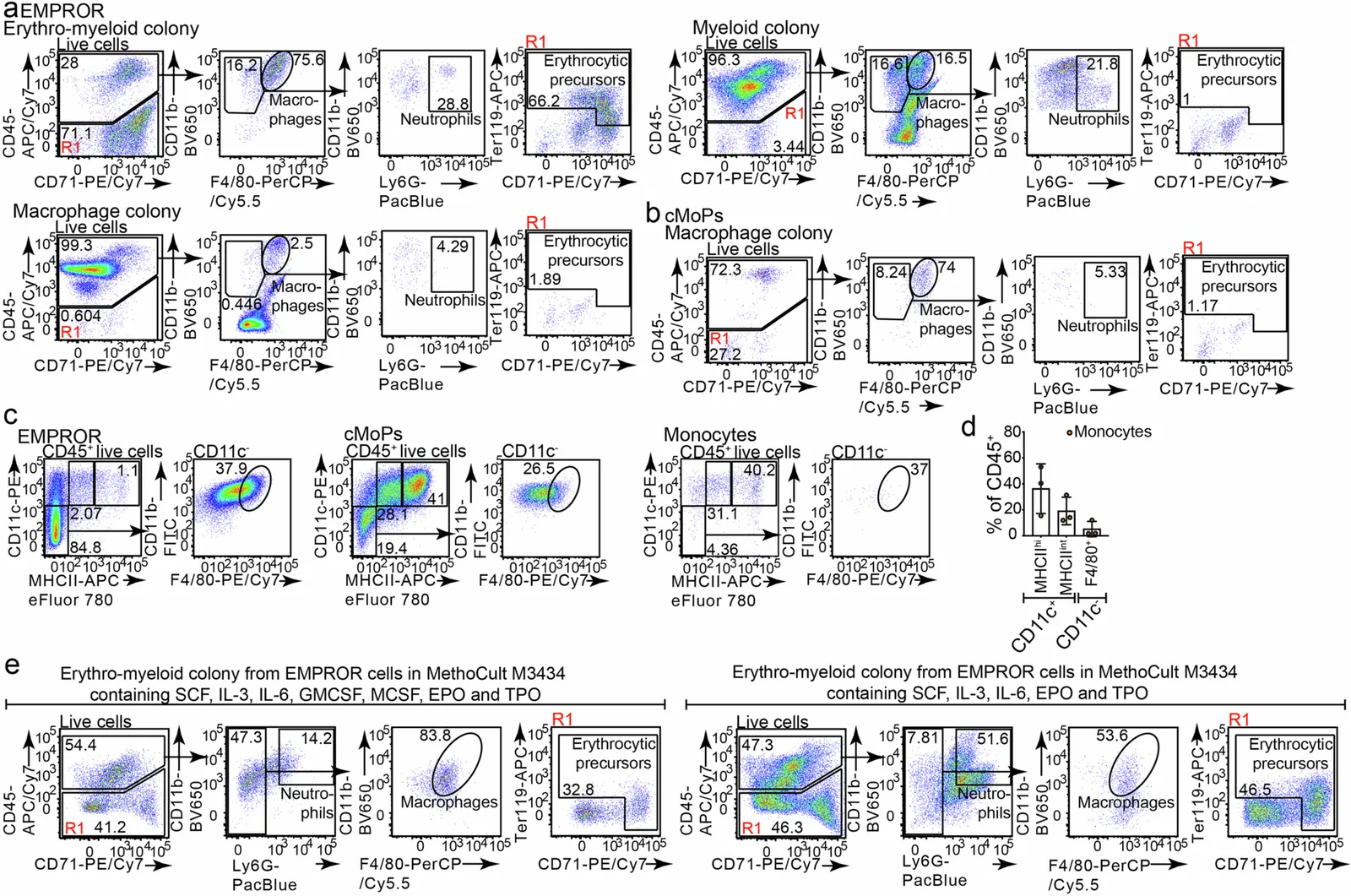
In vitro erythro-myeloid and dendritic cell differentiation potential of EMPROR cells. **a**, Flow-cytometric analysis of colonies obtained post-culture of sorted EMPROR cells in MethoCult™ GF M3434 containing transferrin, SCF, IL-3, IL-6, EPO, and supplemented with TPO, GM-CSF, and M-CSF. Flow plots highlight colonies derived from EMPROR cells with erythro-myeloid, myeloid, and macrophage potential. Erythroid cells were defined as CD45^−^Ter119^+^CD71^+/hi^, neutrophils as CD45^+^CD11b^+/hi^F4/80^−^Ly6G^+,^ and macrophages as CD45^+^CD11b^+^F4/80^+^ cells. Flow plots of three colonies from one of three independent sort and culture experiments are shown. A total of 34 independent colonies were analyzed; **b**, cMoP colonies were restricted to macrophage potential only. Representative flow plots from one of three independent sort and culture experiments are shown; **c**, Flow-cytometric analysis depicting the development of CD11c^+^MHCII^+^ and CD11c^+^MHCII^int^ DCs, and CD11c^−^MHCII^−^CD11b^+^F4/80^+^ macrophages obtained from *in vitro* differentiation of sorted EMPROR cells, cMoPs, and monocytes in the presence of 20 ng/ml GM-CSF. Flow-plots are representative of one of three independent sort and culture experiments; **d**, Quantification of % of CD11c^+^MHCII^+^ and CD11c^+^MHCII^int^ DCs, and CD11c^−^MHCII^−^CD11b^+^F4/80^+^ macrophages obtained from *in vitro* differentiation of sorted monocytes. Data are cumulative of three independent sort and culture experiments. Bars represent mean ± s.d. **e**, Flow-cytometric analysis of colonies obtained post-culture of sorted cell populations in MethoCult™ GF M3434 containing transferrin, SCF, IL-3, IL-6, EPO, and supplemented with TPO, in the presence or absence of GM-CSF and M-CSF. Flow plots highlight colonies derived from EMPROR cells with erythro-myeloid potential in the presence or absence of GM-CSF and M-CSF. Representative flow plots from one of two independent experiments are shown. Source data

To investigate the multilineage potential of EMPROR cells in vitro, we used MethoCult semisolid methylcellulose-based medium, which is formulated to test the progenitor potential of various hematopoietic cells. First, we cultured sorted EMPROR cells in MethoCult containing transferrin, stem cell factor (SCF), interleukin-3 (IL-3), IL-6, erythropoietin (EPO), thrombopoietin (TPO), granulocyte–macrophage colony-stimulating factor (GM-CSF) and macrophage colony-stimulating factor (M-CSF). Under these conditions, EMPROR cells gave rise to colonies comprising macrophage, neutrophil, megakaryocytic and erythroid lineages (Fig. 3d,e, and Extended Data Fig. 8a), establishing that they do indeed possess an erythro–myeloid differentiation potential similar to that described for embryonic EMPs^44^. By contrast, monocytes failed to generate colonies but differentiated into macrophages, whereas cMoP colonies were restricted to the monocyte–macrophage lineage (Extended Data Fig. 8b). Finally, the potential to generate DCs in vitro was assessed^45^. Unlike cMoPs, EMPROR cells failed to generate CD45^+^CD11b^+^CD11c^+^MHCII^hi^ DC-like cells in the presence of GM-CSF, but instead gave rise to CD45^+^CD11b^+^CD11c^−^F4/80^+^ macrophages (Fig. 3f and Extended Data Fig. 8c). Monocytes did not undergo extensive expansion but differentiated into DCs and macrophages (Extended Data Fig. 8c,d). Thus, compared to cMoPs and monocytes, EMPROR cells show distinct lineage potential in vitro, with erythroid, granulocyte and macrophage progenitor activity, while lacking DC or lymphoid potential.

Finally, we wanted to test if EMPROR cells could still retain their EMP potential in the absence of M-CSF and GM-CSF. To this end, we sorted EMPROR cells, cMoPs and monocytes and plated them in semisolid MethoCult M3434 with the relevant cytokine cocktail but in the presence or absence of M-CSF and GM-CSF. In the absence of M-CSF and GM-CSF, both cMoPs and monocytes failed to survive and grow, but EMPROR cells retained their EMP potential, albeit to a lower degree (Fig. 3g,h and Extended Data Fig. 8e). This suggests that M-CSF and GM-CSF are not crucial but potentiate the EMP potential of EMPROR cells.

**Fig. 4:**
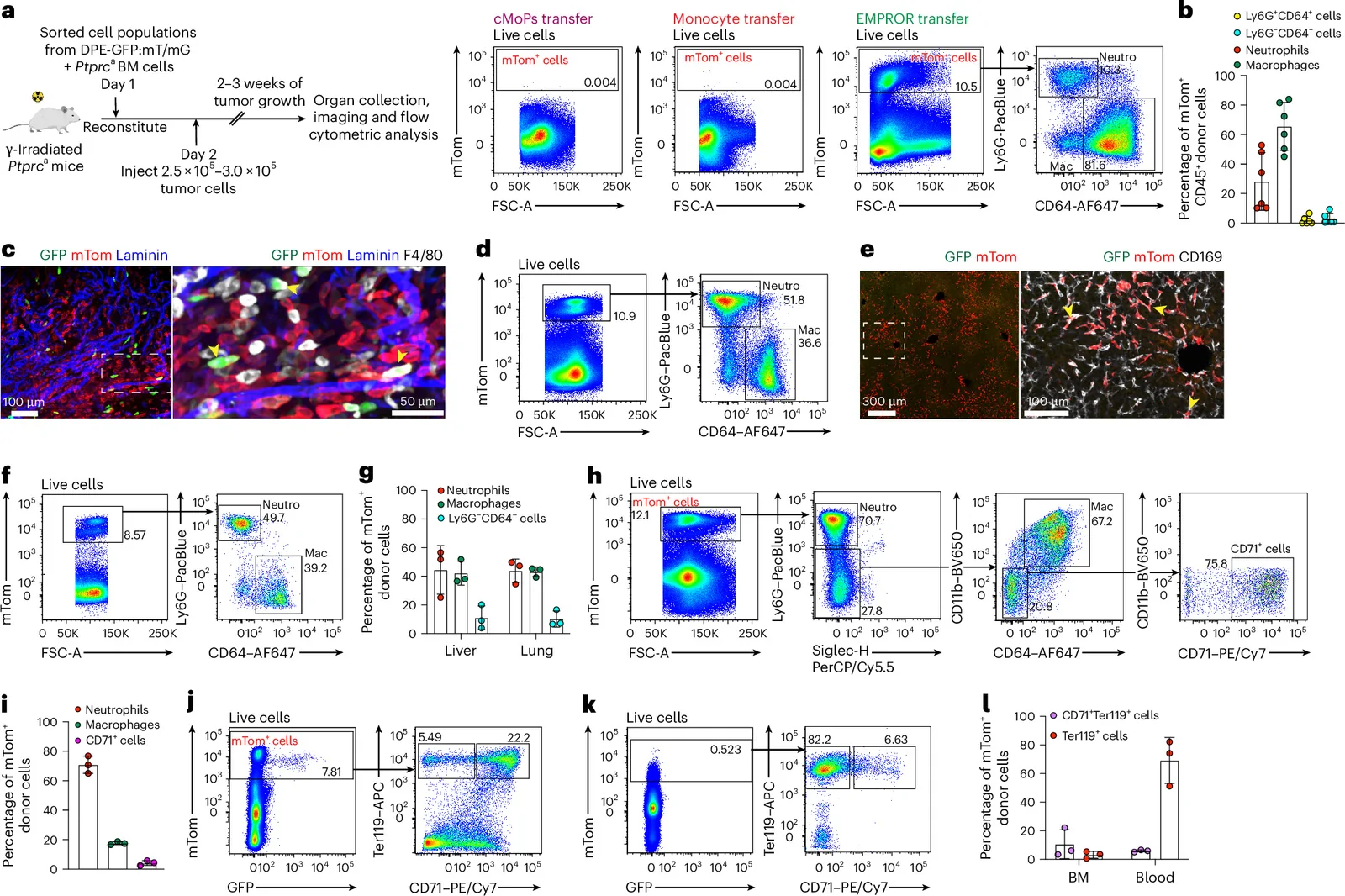
EMPROR cells give rise to erythroid and myeloid cells during tumor development in vivo. **a**, Flow cytometric analysis of E0771 tumors from chimeric mice reconstituted with sorted cMoPs, monocytes or EMPROR cells from DPE-GFP:mT/mG mice. cMoP and monocyte groups show minimal infiltration and expansion of mTom^+^ donor cells, whereas EMPROR cells generate predominantly neutrophils and macrophages. Representative flow plots from one of six independent sort, adoptive transfer and tumor implantation experiments are shown. **b**, Quantification of donor-derived immune populations in tumors. Data are cumulative of six independent sort, adoptive transfer and tumor implantation experiments. Bars represent mean ± s.d. **c**, Confocal imaging of E0771 tumors from EMPROR-reconstituted mice showing mTom^+^, mTom^+^F4/80^+^ and mTom^+^F4/80^+^GFP^+^ donor-derived cells (GFP^+^ cells are marked by yellow arrowheads). **d**, Flow cytometric analysis of liver-infiltrating donor cells showing EMPROR-derived Neu and Mac in tumor-bearing mice. **e**, Confocal microscopy of liver sections from mice reconstituted with EMPROR cells showing mTom^+^CD169^+^GFP^−^ donor-derived Kupffer cells (marked by yellow arrowheads). **f**, Flow cytometric analysis of lungs showing EMPROR-derived mTom^+^ neutrophils and macrophages in tumor-bearing mice. **g**, Quantification of donor-derived immune populations in the liver and lungs of tumor-bearing mice. **h**, Flow cytometric analysis of BM showing EMPROR-derived Neu, Mac and CD71^+^ cells in tumor-bearing mice. **i**, Quantification of myeloid and erythroid populations in the hemolyzed BM of tumor-bearing chimeric mice reconstituted with EMPROR cells. **j**, Identification of erythrocytes and erythrocytic precursors within the non-hemolyzed BM of chimeric mice reconstituted with EMPROR cells. **k**, Identification of erythrocytes and erythrocytic precursors within the non-hemolyzed blood of chimeric mice reconstituted with EMPROR cells. **l**, Quantification of erythrocytes, erythrocytic precursors and reticulocytes within the non-hemolyzed BM and blood of tumor-bearing chimeric mice reconstituted with EMPROR cells. Representative flow plots from one of three independent sort, adoptive transfer and tumor implantation experiments are depicted for **d**, **f**, **h**, **j** and **k**. Data presented for **g**, **i** and **l** are cumulative of three independent sort, adoptive transfer and tumor implantation experiments. Bars represent mean ± s.d. Images for **c** and **e** are representative of tissues collected from tumor-bearing chimeric mice. The data presented are representative of one of three independent sort, adoptive transfer and tumor implantation experiments. Schematic in **a** created in BioRender; Jain, R. https://biorender.com/9z2f7np (2026). Source data

**Extended Data Fig. 9:**
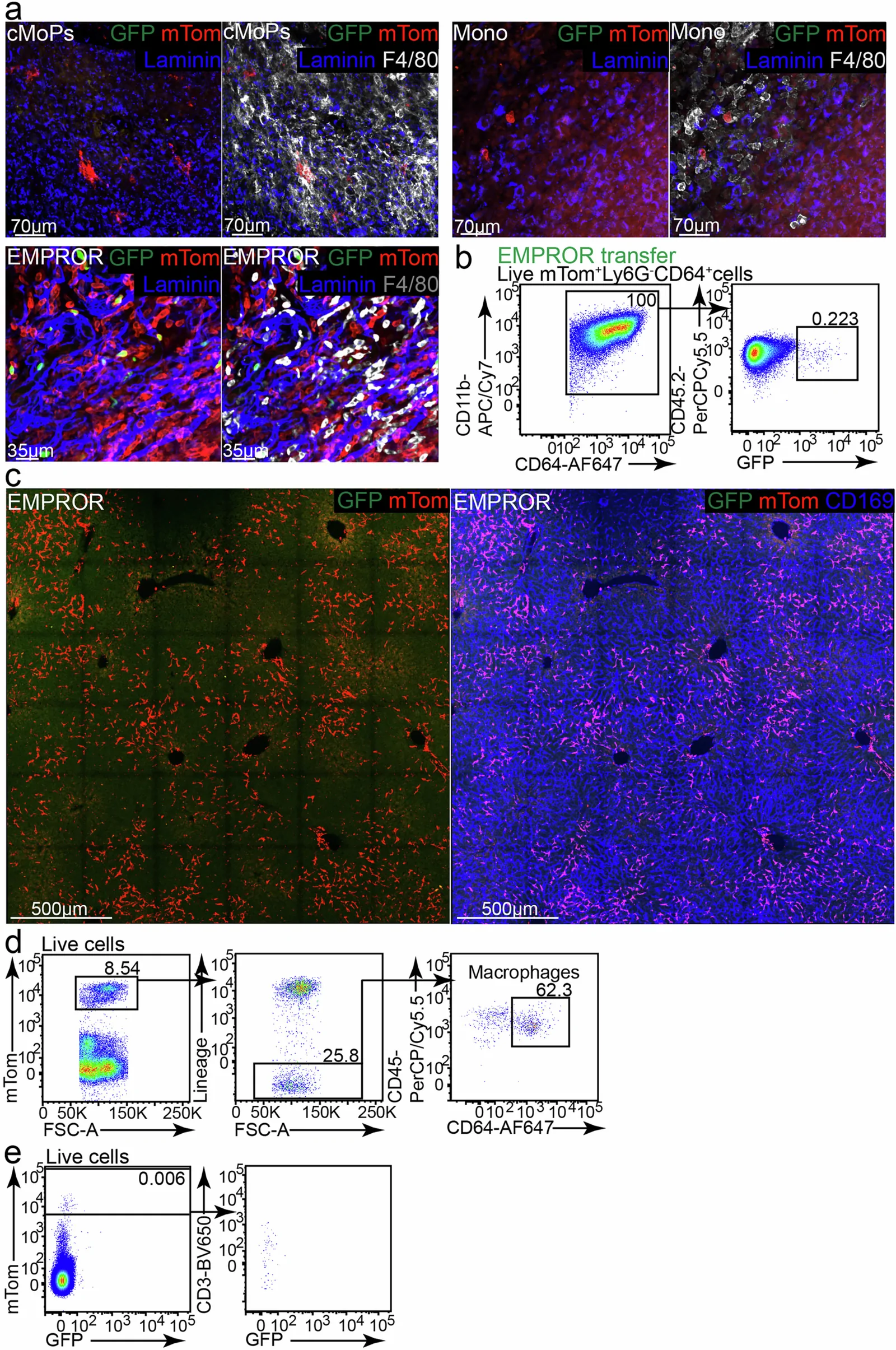
In vivo lineage potential of EMPROR cells. **a**, Confocal imaging of immunostained E0771 tumor sections obtained from mice reconstituted with cMoPs, monocytes, or EMPROR cells shows the presence of mTom^+^F4/80^+^ cells. Images are representative of one of three independent sorts, adoptive transfer and tumor implantation experiments; **b**, Flow cytometric analysis of tumor obtained from chimeric mice reconstituted with EMPROR cells identifies GFP^+^ macrophages within the tumor. Representative flow plots from one of six independent sort, adoptive transfer, and tumor implantation experiments are shown; **c**, Confocal imaging of immunostained liver sections obtained from mice reconstituted with EMPROR cells demonstrates the presence of numerous mTom^+^CD169^+^ cells (large field of view). Images are representative of one of three independent sorts, adoptive transfer, and tumor implantation experiments; **d**, Flow cytometric analysis of brain from chimeric mice reconstituted with EMPROR cells identifies mTom^+^ macrophages within the tissue. Representative flow plots from one of three independent sort, adoptive transfer, and tumor implantation experiments are shown; **e**, Flow cytometric analysis of thymus from chimeric mice reconstituted with EMPROR cells depicts the absence of mTom^+^ T cells within the tissue. Representative flow plots from one of six independent sorts, adoptive transfer, and tumor implantation experiments are shown.

Next, we used our E0771-derived breast cancer model to assess the lineage potential of EMPROR cells in vivo. To faithfully track cells of donor origin arising from adoptively transferred precursors, including erythrocytes, we used DPE-GFP:mT/mG mice (generated by crossing DPE-GFP with mT/mG mice^46^), in which all cells constitutively express a membrane-bound fluorescent reporter (tdTomato; mTom). We engrafted monocytes (5 × 10^5^), cMoPs and EMPROR cells (0.5 × 10^5^–2.5 × 10^5^ each) sorted from the BM of adult DPE-GFP:mT/mG mice into irradiated congenic *Ptprc*^a^ mice. E0771 breast tumors were established 24 h after reconstitution, and various tissues were collected 3 weeks later (Fig. 4a). Transfer of cMoPs and monocytes resulted in scarce mTom^+^ tumor-associated macrophages (TAMs; Fig. 4a and Extended Data Fig. 9a). By contrast, EMPROR cells gave rise to a robust, GFP^−^ TAM population (Fig. 4a–c) and a smaller population of GFP^+^ macrophages (Fig. 4c and Extended Data Fig. 9a,b). In addition, EMPROR cells also yielded neutrophils within tumors (Fig. 4a,b), thereby corroborating their myeloid differentiation potential ascertained in silico and in vitro.

Further analysis of other organs from tumor-bearing mice that had received reporter-tagged EMPROR cells revealed that the livers and lungs harbored a substantial number of mTom^+^ neutrophils and macrophages (Fig. 4d–g and Extended Data Fig. 9c). Confocal microscopic analysis of liver sections identified mTom^+^ macrophages as CD169^+^ Kupffer cells (Fig. 4e and Extended Data Fig. 9c). Additionally, we also collected livers from mice that had received cMoPs and monocytes. Confocal microscopy used to image large fields of view confirmed the presence of mTom^+^CD169^+^ liver macrophages in all three experimental settings (Supplementary Fig. 2), thereby underpinning the ability of monocytes to replete empty macrophage tissue niches.

Furthermore, detailed analysis of other tissues from mice implanted with EMPROR cells highlighted the presence of a small number of donor-derived macrophages in the brain (Extended Data Fig. 9d), while minimal mTom^+^ cells were found in the thymus, reiterating the lack of T cell potential (Extended Data Fig. 9e). EMPROR cells also gave rise to hematopoietic cells in the BM, including neutrophils, macrophages and CD71^+^Ter119^+^ erythroblasts (Fig. 4h–j). Analysis of non-hemolyzed BM and blood showed that circulating CD71^−^Ter119^+^ erythrocytes were also derived from EMPROR cells, thus demonstrating the erythroid potential within the EMPROR population (Fig. 4j–l). Together, these experiments substantiated the erythro–myeloid differentiation potential of EMPROR cells in vivo in the context of a tumor-bearing host.

**Fig. 5:**
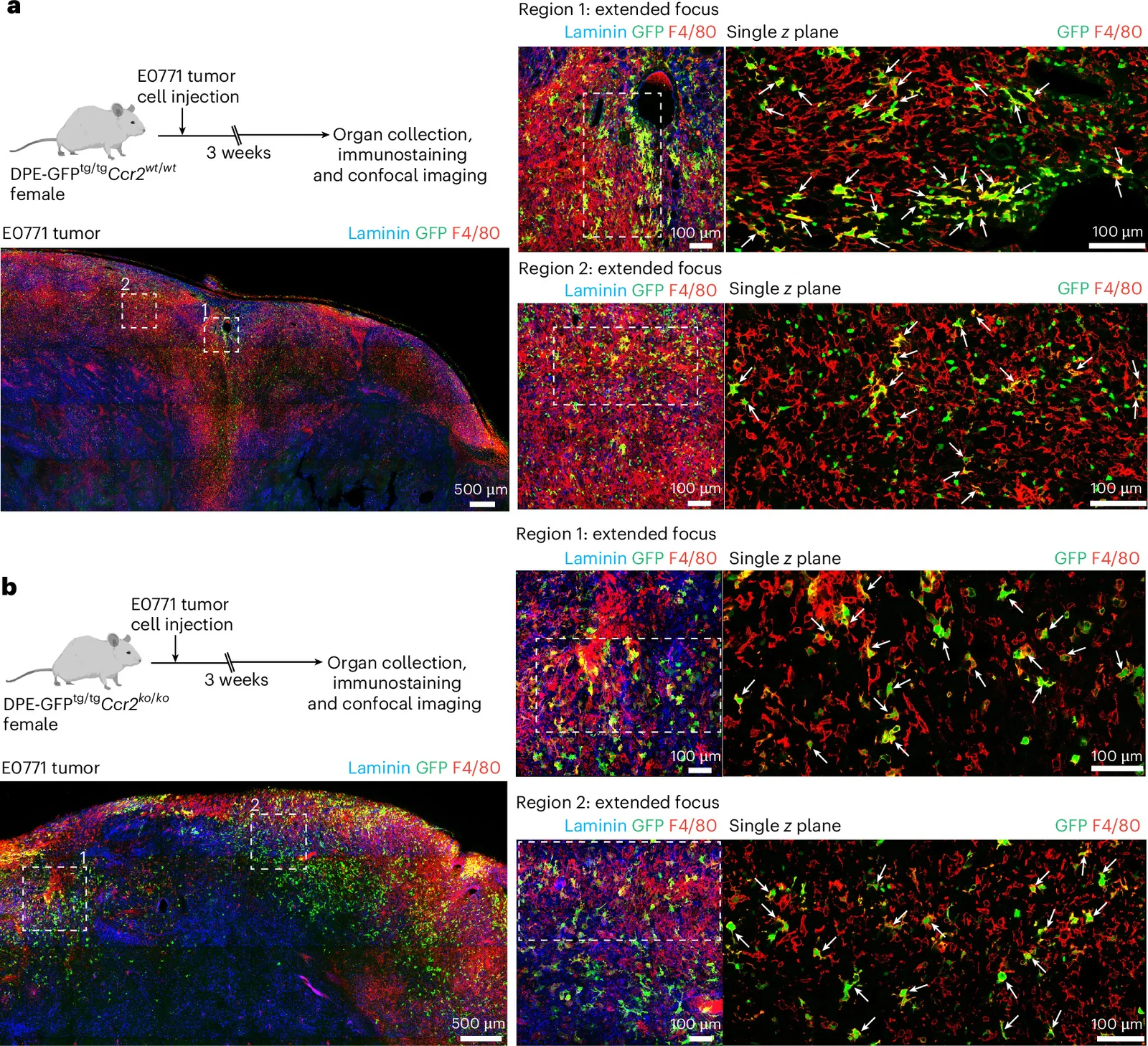
Development of GFP^+^ PVMs in the absence of CCR2. **a**, Confocal imaging of E0771 tumors from DPE-GFP^tg/tg^ *Ccr2*^*wt*/*wt*^ mice. Insets: presence of GFP^+^F4/80^+^ macrophages within the tumor milieu of CCR2-sufficient mice. **b**, Confocal imaging of E0771 tumors from DPE-GFP^tg/tg^ *Ccr2*^*ko*/*ko*^ mice. Imaging of multiple regions shows the presence of GFP^+^F4/80^+^ macrophages within the tumor milieu of CCR2-deficient mice. Images are representative of multiple regions from three independent tumors from three independent mice; ko/ko, knockout. Schematics in **a** and **b** created in BioRender; Jain, R. https://biorender.com/9z2f7np (2026).

Last, we addressed whether tumor-infiltrating GFP^+^ macrophages can arise in the absence of the chemokine receptor CCR2, which would corroborate their development independent of CCR2^+^ monocytes. To this end, we crossed our DPE-GFP mice with *Ccr2*-knockout mice^47^. E0771 breast cancer cells were transplanted in both DPE-GFP × *Ccr2*^−/−^ mice and DPE-GFP × *Ccr2*^wt/wt^ littermates. After 3 weeks, tumors were collected, sectioned and immunostained with anti-F4/80. We saw a robust infiltration of CD4-lineage GFP^+^ PVMs in both *Ccr2*-wild-type and *Ccr2*-knockout mice (Fig. 5a,b). This demonstrates that the CD4 macrophage lineage is capable of developing within tumors in the absence of CCR2.

### Adult EMPROR cells transcriptionally resemble embryonic EMPROR cells and EMPs

**Fig. 6:**
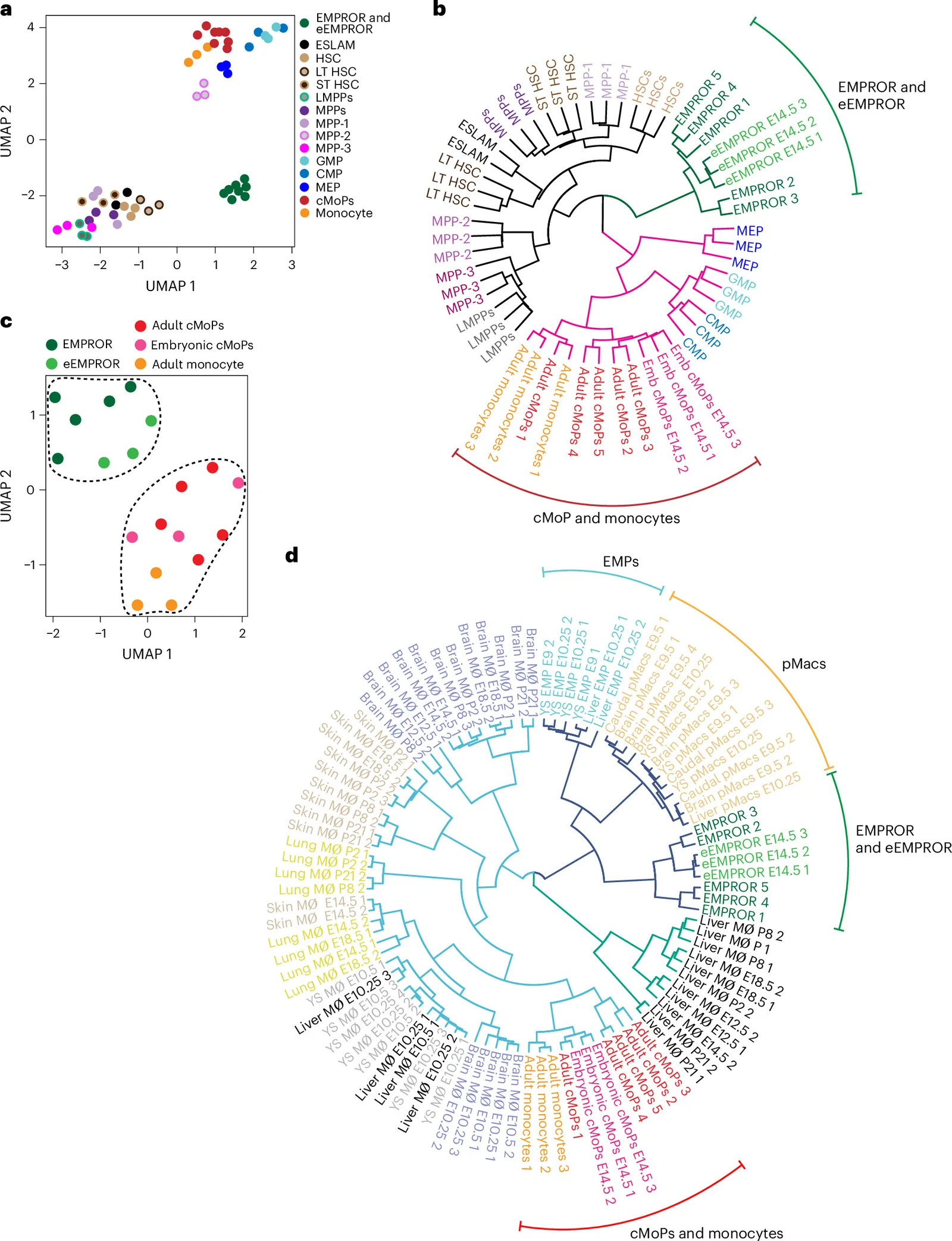
Transcriptional profiling of adult EMPROR and eEMPROR cells. **a**, Comparative analysis of RNA-seq data from adult EMPROR and eEMPROR cells and mouse HSCs and progenitor cells^49^ highlighting the presence of three independent cell clusters within the UMAP. **b**, Dendrogram representation of clustering analysis highlighting the presence of three independent branches consisting of adult EMPROR and eEMPROR cells; HSCs and multipotent progenitors and cMoPs, monocytes, CMPs, GMPs and megakaryocyte–erythrocyte progenitors. **c**, eEMPROR cells and cMoPs were sorted by flow cytometry from pooled fetal livers of DPE-GFP:mT/mG mice at E14.5 (*n* = 6–9 embryos per sort). Monocytes, cMoPs and EMPROR cells were sorted from adult BM. Each symbol is representative of an independent sample. The UMAP plot of bulk RNA-seq data of the individual cell populations shows the close association between eEMPROR and adult EMPROR cells. **d**, Hierarchical clustering of RNA-seq data based on the top 1,000 genes as determined by cell-type-based variance between cell populations described in **c** and classical EMPs, pMacs and pre- and postnatal tissue macrophages. Data for classical EMPs, pMacs and pre- and postnatal tissue macrophages were obtained from a previous study^50^. DESeq2 normalized counts from both studies were combined, scaled and centered. Batch effects were removed using the ComBat batch removal procedure^61^. The data show the clustering of adult EMPROR and eEMPROR cells in proximity to classical EMPs and pMacs^50^. Also note that monocytes and cMoPs cluster with tissue macrophages; Emb, embryonic; ESLAM, EPCR signaling lymphocyte activation molecule; LT HSC, long-term HSC; MΦ, macrophages; MEP, megakaryocyte–erythrocyte progenitor; MPP, multipotent progenitor (four cell populations in total); P, postnatal; ST HSC, short-term HSC; YS, yolk sac.

**Extended Data Fig. 10:**
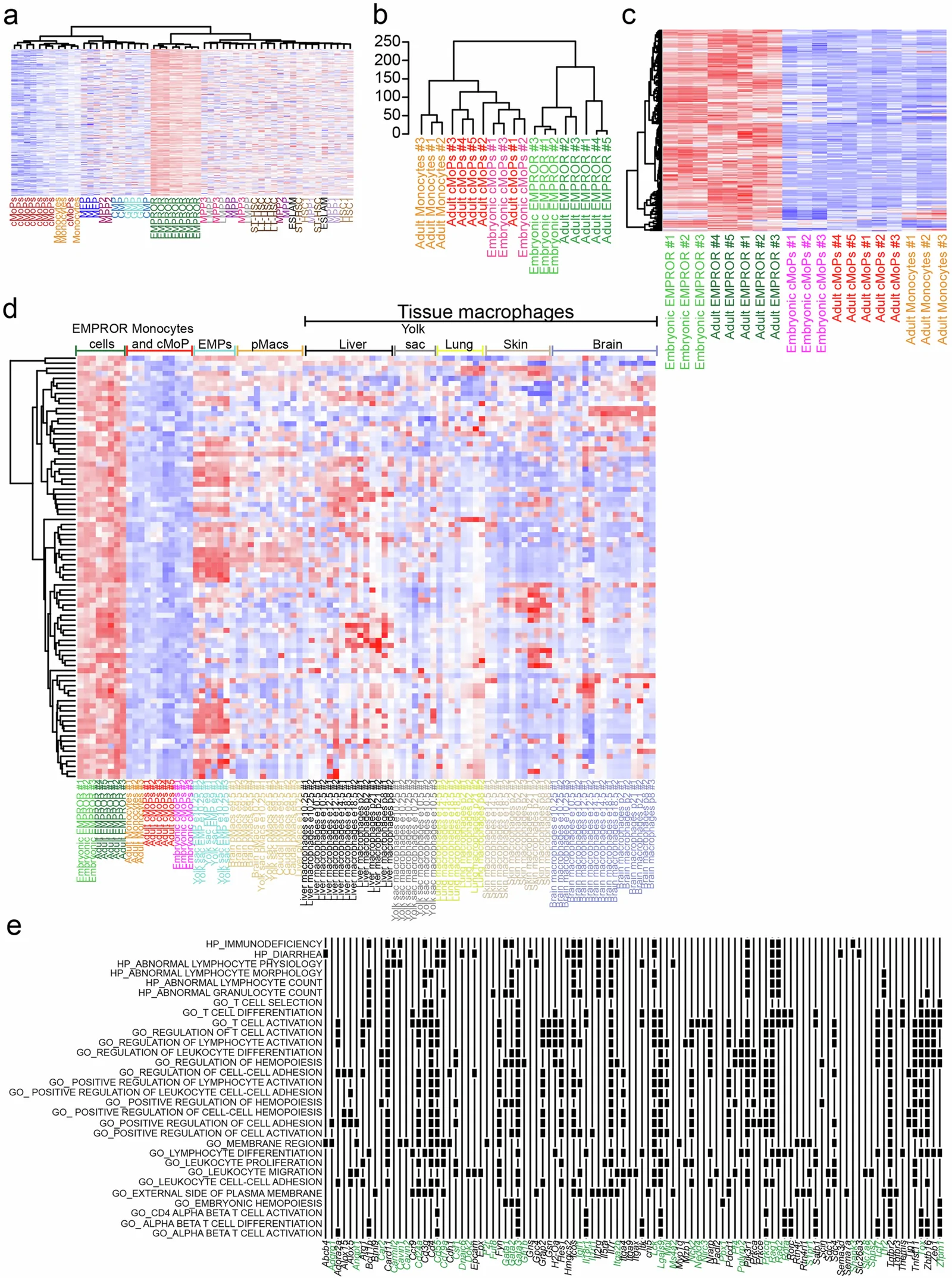
Transcriptional profiling of embryonic and adult EMPROR cells compared to other macrophage precursors and tissue-resident macrophage populations. **a**, Heatmap analysis of the populations highlighting the distinct gene expression within EMPROR cell population when compared to HSCs, and multipotent progenitors, and cMoPs, monocytes and CMP, GMP and MEP^49^; **b**, Hierarchical clustering of bulk RNA-Seq of embryonic EMPROR cells and cMoPs sorted from pooled fetal livers of DPE-GFP:mT/mG mice at E14.5 (n = 6-9 embryos/sort) along with monocytes, cMoPs and EMPROR cells sorted from adult bone marrow; **c**, Heat map displaying the expression profile of the top 100 genes differentially expressed within EMPROR cells when compared to cMoPs and monocytes. To determine the highly expressed genes within these samples, we calculated intra-class correlation coefficients. The top 100 genes displaying the highest intraclass correlation coefficient and higher median gene expression when compared to cMoPs and monocytes were selected for representation; **d**, Heat map depicting the expression of top 100 genes in context to the previously described EMP, pMacs, pre- and post-natal macrophages^50^. Red color indicates high gene expression; blue indicates low gene expression. Columns are color-coded according to the cell type; **e**, Heatmap highlighting the relationship between top100 genes and GO terms. The X-axis shows gene names present in at least one gene set. Genes colored in green were also detected in the previously described Cluster I (Fig. 2k). The y-axis shows gene sets (GO terms). Displayed GO terms have a multiple testing corrected P-value < 0.05. Black squares indicate if a gene is present within gene sets. Note, *Cd4* was present in Cluster IV shared between pDC and EMPROR cells; therefore, it is not highlighted as a common signature between Cluster I (Fig. 2k and Suppl. Table 1). *Abbreviations:* HSC- Hematopoietic stem cells, LT-HSC- Long-term HSC, ST-HSC- Short-term HSC, MPP and MPP1-3- Multipotent progenitors (4 cell populations in total), MEP- Megakaryocyte erythrocyte progenitors, CMP- Common myeloid progenitors, GMP- Granulocyte monocyte progenitors.

In addition to adult EMPROR cells, our studies have highlighted the presence of an embryonic counterpart of EMPROR cells, referred to as ‘eEMPROR’ cells (our companion study)^48^. Using transcriptional profiling, we further tested the relationship between adult EMPROR cells and eEMPROR cells isolated from fetal livers at embryonic day 14.5 (E14.5)^48^ compared to progenitors within the adult HSC lineage (Fig. 6a,b). Comparative analysis of adult EMPROR cells and eEMPROR cells with the ‘pseudo-bulk’ scRNA-seq dataset of mouse HSCs and progenitor cell differentiation^49^ highlighted the presence of three independent cell clusters (Fig. 6a,b). Notably, eEMPROR cells and adult EMPROR cells clustered together to form an independent branch within the dendrogram (Fig. 6b). HSC populations and multipotent progenitors constituted an independent branch. By contrast, cMoPs and monocytes, alongside megakaryocyte–erythrocyte progenitors, CMPs and granulocyte–monocyte progenitors (GMPs; Fig. 6b), formed the third branch. Additionally, EMPROR cells demonstrated the presence of a gene signature distinct from the known mouse HSC and progenitor cell lineages (Extended Data Fig. 10a).

To date, EMPs have only been shown to be present within the embryonic tissues. We tested whether both adult EMPROR and eEMPROR cells share similarities with the transcriptional landscape of previously detailed EMP lineages^50^. To this end, the transcriptional profiles of the isolated eEMPROR cells and cMoPs from fetal livers at E14.5 were compared to monocytes, cMoPs and EMPROR cells collected from adult BM. Uniform manifold approximation and projection (UMAP) analysis combining the first ten principal components revealed that EMPROR cells clustered separately from conventional monocyte lineage cells (Fig. 6c). This was further corroborated by hierarchical clustering analysis, which revealed that EMPROR cells and monocytic cells formed separate branches in the dendrogram (Extended Data Fig. 10b,c).

To determine the relationship of EMPROR cells to other known embryonic macrophage precursors, such as EMPs and pre-macrophages (pMacs), as well as mature macrophage populations in the embryo and adult organism, we performed unbiased comparisons of published transcriptomes^50^. In this large dataset of distinct cell populations, adult EMPROR and eEMPROR cells clustered adjacent to each other and in proximity to other embryonic precursors (Fig. 6d). Embryonic and adult cMoPs, on the other hand, were distinct from EMPROR cells and clustered with other macrophage populations (Fig. 6d). We next identified the top 100 genes most uniformly expressed in adult EMPROR and eEMPROR cell populations. This set of genes also highlighted certain similarities in the gene expression profiles of EMPROR cells, EMPs and pMacs (Extended Data Fig. 10d). Gene Ontology analysis of these top 100 genes depicted enrichment of several biological pathways, including those involved in hematopoiesis and leukocyte development (Extended Data Fig. 10e). The transcriptomic analysis highlights that eEMPROR and adult EMPROR cells exhibit closely related transcriptional programs with close similarities to the previously defined EMPs and pMacs but are distinct from the known adult HSC lineages as well as both adult and embryonic cMoPs.

Together, our experiments reveal a previously unrecognized EMP-like progenitor, EMPROR, within the adult BM. EMPROR cells can replete macrophage niches after depletion or during inflammation and transcriptionally resemble their embryonic counterpart within the developing fetus.

## Discussion

One of the central tenets of macrophage development in the adult is their monocytic origin in inflammation^51^ and cancer^52–54^. Whether additional progenitor lineages contribute toward macrophage production in adulthood is unclear^55–57^. Here we identify and characterize a previously unrecognized adult BM progenitor, EMPROR, with robust erythro–myeloid potential. EMPROR cells are distinct from cMoPs and monocytes by phenotypic marker expression, transcriptional state and lineage potential in vitro and in vivo. Unlike cMoPs and monocytes, EMPROR cells lack DC potential but generate erythroid, neutrophil, megakaryocytic and macrophage lineages. These findings raise the possibility that macrophage replacement or augmentation in injured or inflamed tissues may, under some circumstances, be driven by precursors with an EMP-like program rather than solely by monocyte recruitment.

A central conceptual implication of these findings is that an embryonic erythro–myeloid program, long thought to be developmentally restricted, persists into adulthood. Although EMP-derived macrophages seed tissues before birth and maintain themselves throughout life, bona fide EMP-like progenitors have not been identified in postnatal organisms. Importantly, we use the term ‘EMP-like’ to denote similarity in transcriptional state and lineage potential, without implying direct developmental continuity. EMPROR cells exhibit several properties that converge on an embryonic-like profile: (1) restricted erythro–myeloid lineage potential in vitro and in vivo, (2) transcriptional features reminiscent of early embryonic EMPs and (3) progenitor activity under cytokine-poor conditions lacking M-CSF and GM-CSF, a functional hallmark associated with embryonic progenitors but not typical of adult monocyte-derived progenitors. Together, these data define EMPROR cells as the first identifiable EMP state in the adult BM that functionally and transcriptionally mirrors embryonic EMPs, irrespective of ultimate developmental origin.

Our data also highlight the functional independence of EMPROR-derived macrophages from classical monocyte recruitment. EMPROR cells lack *Ccr2* and *Csf1r*, and in our adoptive transfer experiments, only EMPROR cells, and not monocytes or cMoPs, efficiently generated PVMs. The robust presence of CD4-lineage GFP^+^ PVMs in *Ccr2*^−/−^ tumor-bearing mice further underscores a monocyte-independent macrophage differentiation pathway consistent with EMPROR cell origin. Thus, EMPROR cells provide an alternative route for generating defined macrophage subsets, particularly in settings of inflammation, neoplasia or monocyte insufficiency.

Beyond establishing an alternative macrophage differentiation pathway, our findings suggest that individual macrophage niches may differ in their capacity to recruit distinct precursor populations. EMPROR cells readily generated pial macrophages, a population not efficiently replaced by either GMP- or monocyte DC precursor-derived monocytes^58^. By contrast, GMP-derived monocytes can reconstitute dura mater macrophages following diphtheria toxin-mediated depletion^58^. These observations support a model in which anatomically distinct macrophage niches selectively recruit or support different precursor lineages^58^. Future studies will be needed to dissect how EMPROR- and monocyte-derived macrophages partition among tissue niches and how local cues may shape their relative contribution.

Our integrative transcriptional analyses demonstrate that adult EMPROR cells and their embryonic counterparts share a conserved, EMP-like gene expression program and consistently cluster together, yet remain clearly segregated from HSC-derived progenitors, cMoPs and monocytes, supporting a close developmental relationship between these two populations. This notion is further reinforced by their shared enrichment for erythro–myeloid and hematopoietic gene signatures, as well as by functional data showing that both eEMPROR and adult EMPROR cells display multilineage erythro–myeloid potential reminiscent of embryonic EMPs rather than classical HSC-derived progenitors. Nevertheless, similarity in transcriptional states and functional capacities alone does not formally demonstrate that eEMPROR cells persist as long-lived progenitors into adulthood. A definitive answer to this question will require genetic fate-mapping approaches capable of selectively labeling eEMPROR cells and tracing their progeny into the adult BM. Such experiments are currently limited by the numerical scarcity of eEMPROR cells and by the lack of methodologies that allow for robust precursor labeling without compromising embryo viability. Consequently, we cannot exclude the possibility that adult EMPROR cells arise partially or entirely from HSC-derived progenitors that adopt or re-engage an embryonic-like erythro–myeloid program after birth. Irrespective of their developmental origin, our data establish that the adult BM harbors a progenitor population that transcriptionally and functionally resembles embryonic EMPs.

In summary, our work identifies EMPROR cells as a previously unrecognized, embryonic-like EMP in the adult BM and reveals an alternative, monocyte-independent route to macrophage generation. Whether adult EMPROR cells represent long-lived descendants of embryonic progenitors or an HSC-derived population that re-engages an early developmental program remains an open question, which we have begun to address in our companion study^48^.

The discovery of EMPROR cells provides a new framework for investigating macrophage ontogeny, tissue specialization and heterogeneity and raises the possibility that analogous progenitor populations may exist in humans. Defining their tissue distribution, developmental origin and macrophage-specific functions will be important for understanding their contribution to tissue homeostasis and disease.

## Methods

### Mice

Mouse strains were bred and maintained under specific-pathogen-free conditions (22–26 °C; 12-h light/12-h dark cycle, relative humidity of 55 ± 10%) at the Centenary Institute of Cancer Medicine and Cell Biology or the Core Facility Laboratory Breeding and Husbandry (CFL, Himberg and AKH sites), Medical University of Vienna. C57BL/6J and *Ptprc*^a^ (CD45.1) mice were obtained from the Australian BioResources facility or Animal Resource Centre, Australia. DPE-GFP mice were described previously^27^ (see below). mT/mG mice^46^ were obtained from The Jackson Laboratory, and *Ccr2*-knockout mice^47^ were kindly provided by P. Starkl and S. Knapp (Medical University of Vienna). Mice obtained from other facilities were rested before experimentation. Unless otherwise indicated, mice of either sex aged 6–24 weeks were used; tumor experiments used female mice. All experiments were approved by the relevant animal ethics committees of the Sydney Local Health District, University of Sydney and the Federal Ministry for Women, Science and Research, Republic of Austria, and were performed according to the approved institutional standard operating procedures. Adult mice were killed by CO_2_ asphyxiation or cervical dislocation.

### DPE-GFP mice

DPE-GFP mice were developed by microinjecting a construct containing the distal and proximal CD4 enhancer elements based on the pTG construct described in ref. ^62^. In addition to GFP expression in naive and effector T cells^27,63^, GFP was also expressed by pDCs^39^ and PVMs^25^.

### BM chimera generation

Six- to 8-week-old female *Ptprc*^a^ mice were irradiated (~9 Gy) and reconstituted with 10 × 10^6^ BM cells from female donors. Recipient mice received sulfamethoxazole/trimethoprim (400 mg/80 mg; 0.1 ml per 100 ml of drinking water) for 2 weeks to prevent opportunistic infections. For experiments using transgenic *Rag1*-deficient donor BM, 1% of the total, that is, 10 × 10^4^ of either C57BL/6J or *Ptprc*^a^-derived BM cells, were co-injected with the 10 × 10^6^ transgenic *Rag1*-deficient donor cells to restore the adaptive immune cell lineages.

### Breast cancer cell implantation

To generate mammary tumors, 0.25–0.5 × 10^6^ E0771 cells (breast adenocarcinoma) were injected into the inguinal mammary fat pad of virgin female mice (8–12 weeks old). Tumors typically become palpable within 7–10 days and reach the endpoint volume (~0.9 cm^3^) within 2–3 weeks. All ethics permits allowed us to grow tumors up to 1 cm^3^ in volume (calculated using the formula *V* = 0.5 × length × width^2^). The experiments were performed at 0.8–0.9 cm^3^ tumor volume.

### Flow cytometric analysis and phenotyping of BM precursors

BM cells were collected from the femurs and tibiae of 6- to 12-week-old DPE-GFP or DPE-GFP:*Rag1*^−/−^ mice, flushed into FACS wash (1× PBS + 2% FBS + 2mM EDTA + 0.01% sodium azide), filtered through a 70-μm cell strainer and centrifuged at 524*g* for 5–10 min at 4 °C. Red blood cells (RBCs) were lysed with ACK buffer, and the cells were neutralized with FACS wash, filtered through a 70-μm cell strainer and centrifuged at 524*g* for 5–10 min at 4 °C. EMPROR cells were identified using lineage markers (CD3e, Ly6G, NK1.1, CD19 and Siglec-H; PerCP/Cy5.5), CD115 (APC), CD117 (PE/Cy7) and CD135 (APC/Cy7). For additional phenotyping, markers of interest were assigned to available PE, BV421, Pacific Blue or APC channels, with CD115 or CD117 fluorophores adjusted where required. Fluorescence minus one and isotype controls were used to define marker expression. Antibodies, clones and fluorophores are listed in Supplementary Table 3.

For initial identification and phenotypic characterization of EMPROR cells, including assessment of mature macrophage marker expression, CD64 was excluded from the lineage cocktail (Fig. 2a–d and Extended Data Fig. 3). Thereafter, CD64 was included in the lineage cocktail in all subsequent experiments to exclude mature macrophages.

### Sorting of BM precursor populations for various assays

BM from femurs, tibiae and spines of two to seven female DPE-GFP, DPE-GFP:*Rag1*^−/−^ or DPE-GFP:mT/mG mice (6–16 weeks old) was mechanically dissociated in sort buffer (1× HBSS + 2% FBS + 0.2% EDTA), filtered through a 70-μm cell strainer and centrifuged at 524*g* for 10 min at 4 °C. RBCs were lysed with ACK buffer, and samples were neutralized and washed with sort buffer. Cells were incubated with Fc block and biotinylated CD135 for 45–60 min on ice, washed and stained with lineage markers (CD3e, Ly6G, NK1.1, CD19, Siglec-H and CD64; PerCP/Cy5.5), CD115 (APC), CD117 (PE/Cy7) and streptavidin–APC/Cy7 (to mark CD135 expression). Cells were sorted on a BD FACSAria II into FBS. Only populations displaying >95% postsort purity were used.

### RNA extraction

BM precursor populations as described above were directly sorted into RLT Plus (lysis) buffer provided with the Qiagen RNeasy Plus Micro Kit. RNA was extracted using the Qiagen RNeasy Plus Micro Kit (Qiagen) according to the manufacturer’s instructions.

### RT–qPCR analysis

RNA was isolated from sorted populations as described above. cDNA was generated using random hexamers and M-MLV reverse transcriptase. RT–qPCR was performed using the SensiFAST Probe Lo-ROX Kit (Bioline) on a Stratagene Mx3005P. Relative expression was normalized to *Gapdh* and *Actb* and calculated using the ${2}^{-\Delta \Delta {C}_{t}}$ method. Three biological replicates were analyzed per gene, with statistical comparisons performed by one-way ANOVA with a Tukey’s multiple-comparison test. TaqMan assays and assay identifiers are listed in Supplementary Table 4.

### Meningeal macrophage depletion and adoptive transfer of sorted precursors

Meningeal macrophages were depleted using mannosylated clodronate liposomes (Clodrosome, Encapsula Nanoscience). Mice were anesthetized with ketamine/xylazine (100 mg per kg (body weight)/10 mg per kg (body weight)), and 10 μl of liposomes was administered into the subarachnoid space by i.t. injection through the parietal bone. A second 10-μl dose was administered 24 h later. After 3–4 days, sorted immune cell precursors in 10 μl of HBSS were administered via the same route. Mice recovered for 4–6 weeks before intravital imaging.

### Surgical preparation for intravital imaging

#### Mammary fat pad and tumor imaging

Mammary fat pad and tumor imaging was performed as described previously^64^. Briefly, mice were anesthetized with ketamine/xylazine (100 mg per kg (body weight)/10 mg per kg (body weight)), with repeated half-dose injections as required, and maintained on a temperature-controlled surgical platform. Adequate depth of anesthesia was monitored regularly by assessing hind leg withdrawal and the palpebral reflex. Following hair removal, a lateral skin incision was made, and the skin was separated from the underlying tissue using blunt dissection. The skin flap was secured with sutures and superfused with buffered saline (pH 7.4; at 37 °C). Exposed tissues were maintained at 37 °C. Evans Blue conjugated to serum albumin was administered intravenously to visualize blood vessels.

#### Brain imaging

Mice were anesthetized with ketamine/xylazine (100 mg per kg (body weight)/10 mg per kg (body weight)) and received buprenorphine (0.05–0.1 mg per kg (body weight)). After immobilization of the head, the cranial bone was exposed, and a 4- to 5-mm region thinned to approximately 8–15 µm using a dental drill under regular saline irrigation, avoiding damage to the meninges. Evans Blue–albumin was administered intravenously to visualize the vasculature. Where indicated, 10 µl of TRITC–dextran was injected into the subarachnoid space 4–6 h before cranial window preparation.

### Intravital imaging

Multiphoton microscopy was performed using a LaVision BioTec TriMScope equipped with a ×20/0.95-NA water-immersion objective, six external non-descanned dual-channel reflection/fluorescence detectors, a diode-pumped, wideband modelocked Ti-Sapphire femtosecond laser (Mai Tai HP; SpectraPhysics, 720–1,050nm, <140 fs, 90 MHz) and an APE optical parametric oscillator system (tuning range 1,050–1,400 nm). Images were acquired over 270 × 270 μm fields at a resolution of 500 × 500 pixels with six to eight *z* steps for durations specified by the experimental design.

### Confocal imaging

Confocal laser-scanning microscopy was performed on tissue sections or whole mounts as per previously established protocols^25^. Briefly, tissues were fixed overnight in 4% paraformaldehyde at 4 °C and washed in immunostaining buffer (PBS, 2% fetal calf serum (FCS), 2 mM EDTA, 0.2% sodium azide, 0.3% Triton X). Mammary fat pads were stained as whole mounts; tumors, lungs and adult livers were embedded in 5% low-melting agarose and sectioned at 100–200 µm using a Vibratome 1000Plus. Collected and fixed brain tissues were directly mounted on the Vibratome 1000Plus and sectioned. Samples were incubated with primary antibodies for 48–72 h at 4 °C, washed and incubated with secondary antibodies overnight at 4 °C. Antibodies and dilutions are listed in Supplementary Table 5. Samples were mounted in DABCO and imaged using a Leica TCS SP5 or Olympus FV3000 confocal microscope. Images were analyzed using Volocity (Perkin Elmer) and Fiji (Image J^65,66^; imagej.net/ij).

### Lineage potential assays

#### B and T cell potential

##### OP9-DL1 system

B and T cell potential was assessed using OP9 and OP9-DL1 coculture, respectively, with minor modifications to published protocols^42^. OP9/OP9-DL1 cells were maintained in MEM (Sigma) containing 20% FBS, 2% penicillin/streptomycin, 1% L-glutamine and β-mercaptoethanol and were 80–90% confluent at initiation of coculture. Progenitors were sorted from the BM of four 6- to 12-week-old female DPE-GFP mice as described above. CD117^+^Sca-1^+^Lin^−^ (CD3e, CD4, CD11b, CD19, Siglec-H, Ly6G, NK1.1) cells served as a positive control. Sorted cells were seeded on top of 80–90% confluent OP9 cells containing 5 ng ml^−1^ FLT-3L (R&D) and 1 ng ml^−1^ IL-7 (PeproTech).

For T cell differentiation, cells were passaged onto fresh OP9-DL1 cultures on days 5, 8 and 12 and analyzed by flow cytometry on day 16. For B cell differentiation, cells were passaged onto fresh OP9 cultures on days 5, 8, 12 and 16 and analyzed on day 20. At each passage, 25% of EMPROR cells, cMoPs and monocyte cultures and 10% of positive-control cultures were transferred to account for their differing proliferation rates.

#### B cell development: MethoCult

BM was collected from the femurs, tibiae and spines of three 6- to 12-week-old female DPE-GFP mice. For B cell development, 10,000 sorted cells were plated in 1 ml of MethoCult M3630 (STEMCELL Technologies) containing 2% penicillin/streptomycin. After 7 days, colonies were collected, washed and analyzed by flow cytometry. Total BM cells (2 × 10^5^) cultured under identical conditions served as positive controls.

#### Macrophage potential: MethoCult and liquid culture

For macrophage differentiation in semisolid medium, 1,000 sorted cells were cultured in 1–2 ml of MethoCult GF M3434 (STEMCELL Technologies) supplemented with 50–100 ng ml^−1^ M-CSF (PeproTech). After 7 days, colonies were collected, washed and analyzed by flow cytometry. Alternatively, sorted cells were cultured in DMEM GlutaMAX containing penicillin/streptomycin, sodium pyruvate, 10% FCS and 50 ng ml^−1^ M-CSF, or Complete RPMI media with 50 ng ml^−1^ M-CSF. Differentiated adherent cells were imaged in situ or collected for flow cytometry.

#### Erythro–myeloid potential: MethoCult

BM cells were collected from the femurs, tibiae and spines of three 6- to 12-week-old female DPE-GFP mice (as described above). EMPROR cells, cMoPs and monocytes (800–1,000 cells per replicate) were cultured in duplicate in 1.5 ml of MethoCult GF M3434 (STEMCELL Technologies) containing transferrin, SCF, IL-3, IL-6 and EPO and supplemented with 5 ng ml^−1^ TPO (PROSPEC Biotech), 3 ng ml^−1^ GM-CSF (PROSPEC Biotech) and 5 ng ml^−1^ M-CSF (PeproTech). The cell suspension was plated in two 35-mm Petri dishes and incubated at 37 °C for approximately 2 weeks.

We obtained 20–30 distinct colonies for each of the EMPROR cell and cMoP groups. Ten to 20 colonies per group per experiment were analyzed using flow cytometry, while 5–10 colonies per group per experiment were used for Giemsa staining and cytological evaluation. For monocytes that form three to four cell clusters, the complete plate containing MethoCult was analyzed by flow cytometry, Giemsa staining and cytological evaluation.

The 20 colonies used for cytology, detailed in the figure legends, are colonies that were successfully picked, washed free of MethoCult, cytospun on glass slides, stained and imaged. These data exclude colonies that were picked but either had very few cells or staining and imaging artifacts were present.

#### DC potential

Sorted cell populations were cultured as described previously^45^ in RPMI 1640 supplemented with glutamine, penicillin, streptomycin, 2-mercaptoethanol, 10% FCS and 20 ng ml^−1^ GM-CSF (ProSpec) at 37 °C and 5% CO_2_. Differentiated cells were collected for flow cytometric analysis.

### Giemsa staining and cell cytology

MethoCult colonies were collected, washed and cytocentrifuged onto glass slides for 5 min at 28.2*g* using a Cytospin 3 cytocentrifuge (Shandon). Slides were air dried overnight, fixed in methanol for 5 min and stained with Giemsa (Sigma-Aldrich) for 30 min. Slides were then washed, dried and imaged using a Leica DM6000B microscope with a ×20 objective. Tiled images encompassing the complete cytospin area were assessed for the presence of different cell lineages.

### Assessment of in vivo lineage potential

Female *Ptprc*^a^ mice (6–8 weeks old) were irradiated (~9 Gy) 1 day before sorting of the precursors. On the day of the sort, BM precursors were sorted from femurs, tibiae and spines of three to six female DPE-GFP:mT/mG mice (6–17 weeks old) as described above. Sorted cells were combined with 2 × 10^6^–5 × 10^6^ *Ptprc*^a^-derived BM cells and adoptively transferred into irradiated recipients. One day later, recipients were injected with 2.5 × 10^5^–3.0 × 10^5^ E0771 breast adenocarcinoma cells into the inguinal mammary fat pad. Mice were killed when tumors approached 0.8 cm^3^, and donor-derived cells were assessed in the indicated tissues as follows.

### BM

Femurs and tibiae were flushed into FACS wash and filtered, and RBCs were lysed with ACK lysis buffer before staining for flow cytometry. ACK lysis was omitted when assessing donor contribution to the erythroid lineage.

### Brain

Brain tissue was collected as per previously established protocols^67^ with modifications. Briefly, brains were mechanically dissociated in 1× PBS containing 10% FBS, 1 mg ml^−1^ collagenase type IV and 2% (vol/vol) DNase, homogenized using an 18-G needle and digested for 40 min at 37 °C. Digests were filtered through a 100-μm strainer, washed and enriched using 30% Percoll (GE Healthcare; 931*g*, 8 min, 4 °C), followed by brief ACK lysis and washing before flow cytometry.

### Liver

Livers were perfused in situ with 1× PBS containing 10% FBS and 1 mg ml^−1^ collagenase IV and dissociated in 1× PBS containing 10% FBS, 1 mg ml^−1^ collagenase type IV and 2% (vol/vol) DNase for 40 min at 37 °C. Digests were filtered through 100-μm strainers, washed (524*g* for 5 min at 4 °C) and enriched using 40% Percoll (931*g*, 8 min, 4 °C), followed by ACK lysis, washing and staining for flow cytometry.

### Skin

Cutaneous tissue was processed as described previously^25^. Briefly, ears were split into dorsal and ventral halves, minced and digested for 1 h at 37 °C in 1× PBS containing 10% FBS, 5 mg ml^−1^ collagenase type IV and 2% (vol/vol) DNase. Digests were filtered, processed for RBC lysis and washed before staining for flow cytometry.

### Whole blood and blood leukocytes

Blood obtained by cardiac puncture (after CO_2_ asphyxiation) was collected into 500 μl of Alsever’s solution. An aliquot was stained directly for analysis of whole blood. The remaining cells were processed for RBC lysis with ACK buffer before leukocyte analysis.

### Thymus and lung

Tissues were minced, passed through a 70-µm strainer and washed. ACK lysis was performed when visually required before flow cytometric staining.

### Tumors

Tumors were minced and digested for 1 h at 37 °C in PBS containing 10% FBS, 1 mg ml^−1^ collagenase type IV and 2% (vol/vol) DNase. Digests were filtered, washed and centrifuged at 524*g* for 5 min at 4 °C. RBCs were lysed with ACK lysing buffer, followed by neutralization with FACS wash and centrifugation at 524*g* for 5 min at 4 °C. Cells were layered over 3 ml of Ficoll. Following centrifugation at 931*g* for 1 h at 22 °C with brakes off, the Buffy layer was collected, washed, stained and analyzed by flow cytometry.

### Sorting of BM cells for single-cell sequencing

BM cells from the femurs, tibiae and long bones of five 10- to 12-week-old DPE-GFP mice were processed as described above. The resulting cell suspension was distributed into three tubes to sort EMPROR cells and GFP^+^ and CD45^+^ cells. EMPROR cells were identified using lineage markers (CD3e, Ly6G, NK1.1, CD19, Siglec-H and CD64; PerCP/Cy5.5), CD115 (APC), CD117 (PE/Cy7) and CD135 (APC/Cy7). CD45^+^ cells were identified using CD45-PerCP/Cy5.5. Unstained aliquots were used to sort pan-GFP^+^ and total BM cells. DAPI was used for dead cell exclusion. Cells were sorted at high purity using a BD FACSAria Fusion directly into 1× PBS with 0.08% BSA.

A total of 4,500 EMPROR cells, 30,000 GFP^+^ cells and 15,000 CD45^+^ cells were collected. The CD45^+^ fraction was supplemented with 15,000 total BM cells to capture CD45^−^ populations, including erythroid and megakaryocytic lineages.

### Single-cell sample preparation and sequencing

Sorted cells were processed using the 10x Genomics Chromium platform and 5′ V2 protocol. Libraries were quantified using Qubit (Invitrogen, Q33231) and assessed using TapeStation high-sensitivity D5000 and D1000 (Agilent, 5067-5592 and 5067-5582, respectively) before sequencing on an Illumina NovaSeq 6000 at the Biomedical Sequencing Facility at the CeMM Research Center for Molecular Medicine. Reads were demultiplexed using bcl2fastq v2.20.0.422 and processed using Cell Ranger (v7.0.0, 10x Genomics) against the mm10 mouse genome. Libraries yielded an average of 106 million reads with an average of 40,000 reads per cell. Doublets were identified using the scran package^68^ (Bioconductor) by comparison with simulated doublets. Doublet scores were calculated using 200 nearest neighbors, and cells with scores of >4 were excluded independently from each dataset.

### Analysis of single-cell data

Following sample-level quality control, raw read counts were combined and analyzed using Seurat. Pearson residuals generated using sctransform^69^ were used for PCA and differential expression analysis with sequencing depth, mitochondrial RNA content and batch included in the regression model. Principal components 1–8 were used for nearest neighbor and *t*-SNE analyses, and cluster-specific markers were identified using Wilcoxon rank sum tests. RNA velocity analysis was performed using scVelo^38,70^ based on relative spliced and unspliced transcript abundance.

### Sorting of precursors from E14.5 livers

E14.5 DPE-GFP:mT/mG livers were mechanically dissociated, passed through a 19-G needle, filtered through a 100-μm cell strainer, centrifuged at 754*g* for 10 min at 4 °C, processed for RBC lysis with ACK lysing buffer for 5–10 min at 37 °C, neutralized with FACS wash, centrifuged and stained for sorting using a BD FACSAria II. Samples displaying >95% purity were used for bulk RNA-seq.

### Bulk RNA-seq of adult BM precursors

RNA quality was assessed using an Agilent 2100 Bioanalyzer. High-quality RNA from three independent experiments was used for library preparation and sequenced on an Illumina HiSeq 2500 platform using 100-base pair single-read sequencing, with a minimum of 10 × 10^6^ reads per sample achieved. Data handling, sequence alignment and assembly were performed in collaboration with the Australian Genome Research Facility’s bioinformatics facility.

### RNA-seq for embryonic precursors

RNA-seq was performed at the Ramaciotti Centre for Genomics (University of New South Wales). RNA quality was assessed using an Agilent 2100 Bioanalyzer. Two nanograms of high-quality RNA from three independent experiments was used as an input for SMARTer Total stranded mammalian pico v2 library preparation. Libraries were run using the Illumina NextSeq 500 2 × 75 base pair HO flowcell platform and paired-end sequencing. Two additional adult progenitor samples were included as controls.

### Hexagonal plots and rose diagrams

RNA-seq quality was assessed using FastQC^71^. FASTQ files were aligned to the *Mus musculus* reference genome grc38 using RSEM^72^ and default parameters. Hexagonal plots were generated with the Triwise package in R^18,73^, using recommended parameter settings in the package vignette. Gene expression read counts were normalized using the R package edgeR^74^. Differentially expressed genes were obtained by applying the same pipeline used in the Triwise vignette, which is based on the Limma R package^75,76^. Black dots represent differentially expressed genes, and gray dots represent other genes in the dataset. Each of the 120° angles is associated with one of the three populations, and the distance from the center is proportional to the fold change difference. For each gene module, differentially expressed genes are shown as red dots in the hexagonal plots (differentially expressed genes associated with the gene set).

### Two-dimensional PCA, volcano plots, *k*-means clustering and Gene Ontology terms

For gene expression analysis of RNA-seq data (HSCs and LMPPs from GSE76140 (ref. ^40^), CMPs from GSE107495 (ref. ^41^) and the manuscript dataset), the adaptors and low-quality bases assessed using FastQC^71^ 0.11.8 were trimmed by Trimmomatic^77^ 0.38 with the default settings. Trimmed reads were then aligned to the Ensembl 86 (GRCm38.p4) reference genome using STAR^78^ 2.5.2a. FeatureCounts^79^ 1.5.1 was subsequently used to convert aligned short reads into read counts for each sample. The data were then analyzed using R^80^ 3.1.2 and DESeq2 (ref. ^81^) 1.28.1. ComBat^59,60^, based on the empirical Bayes method, was applied to the values normalized by variance-stabilizing transformation from DESeq2 for the batch effect adjustment between samples. Differentially expressed genes of various groups were identified using the Wald test^82^, with a fold change of >2, *P* of <0.05 after Benjamini–Hochberg^83^ correction and base mean of >1. Unsupervised *k*-means clustering analysis^84^ was performed using the significantly differentially expressed genes. Gene Ontology analysis was performed using the enrichGO function in the clusterProfiler R package^85^. *P* values for enrichment were calculated using a hypergeometric test with Benjamini–Hochberg correction. PCA^86^ plots and heat maps were generated in R.

### Integration with publicly available datasets

The dataset GSE81686 comprises EMPs, pMacs and macrophages taken from different tissues and at specific time points during embryonic and early development^50^. We downloaded the DESeq2 normalized count matrix from the GSE81686 dataset to serve as input for this analysis. Briefly, data were aligned with STAR, features were counted with feature count, and counts were normalized with DESeq2. We created a combined dataset consisting of 13,190 EntrezIDs present in both studies across 95 samples. The combined dataset was scaled and centered. The batch effect introduced by two independent RNA-seq analyses in the present study, combined with the published dataset, was removed using the ComBat batch removal procedure^61^. Using linear mixed models^87^, we decomposed the variance for every gene in the dataset into variance introduced by batch, experiment (that is, GSE81686 and the manuscript datasets), age (that is, embryonic, postnatal and adult) and cell type. For PCA, we used the top 1,000 genes explaining the most variance accounted for by cell type (Supplementary Table 2). These genes were used as input for PCA. We used the first ten principal components for hierarchical clustering and UMAP visualization of the integrated dataset. Similar methodology was used for the integration of datasets for comparative analysis of adult EMPROR and eEMPROR cells with the ‘pseudo-bulk’ scRNA-seq dataset of mouse HSC and progenitor cell differentiation^49^.

### Genes uniformly expressed in adult EMPROR and eEMPROR cells

To define a set of genes highly expressed in both eEMPROR and adult EMPROR cells, we calculated single-score intraclass correlation coefficients for each gene. For this, we grouped the data into EMPROR cells (consisting of adult and embryonic), cMoPs (consisting of adult and embryonic) and monocytes. We used the CRAN package ‘irr’ and defined a two-way random model (model 2), evaluating the consistency within each group. The top 100 genes with the highest intraclass correlation coefficients, as well as a higher median gene expression than cMoPs and monocytes, were selected for heat map representation (Supplementary Table 2). Over-representation analysis was performed as described in the previous sections.

### Cell lines used in this study

E0771 cells were a kind gift from R. Anderson (Olivia Newton-John Cancer Research Institute). OP9/DL1 cells were a kind gift from S. Naik (Walter and Eliza Hall Institute of Medical Research). None of the cell lines used in this study are on the list of misidentified cell lines maintained by the International Cell Line Authentication Committee.

### Statistical analysis

Statistical analyses were performed using Prism software (GraphPad Software). A one- or two-way ANOVA followed by a Tukey’s or Šidák multiple-comparison test was used as indicated in the figure legends or individual sections in the Methods. A *P* of <0.05 was considered statistically significant. No statistical methods were used to predetermine sample sizes, but our sample sizes are similar to those reported in previous publications. Data distribution was assumed to be normal, but this was not formally tested. The experiments were not randomized. Data collection and analysis were not performed blind to the conditions of the experiments.

### Reporting summary

Further information on research design is available in the Nature Portfolio Reporting Summary linked to this article.

## Online content

Any methods, additional references, Nature Portfolio reporting summaries, source data, extended data, supplementary information, acknowledgements, peer review information; details of author contributions and competing interests; and statements of data and code availability are available at https://doi.org/10.1038/s41590-026-02686-1.

## Supplementary information

Supplementary Information Supplementary Figs. 1 and 2 and Tables 3–5.

Reporting Summary

Supplementary Table 1 Differentially expressed genes in each cluster (Fig. 2k). The tab for cluster I depicts the Gene Ontology list (Fig. 2i, highlighted in yellow).

Supplementary Table 2 Gene list for data depicted in Extended Data Fig. 10.

Supplementary Table 3 Antibodies used for flow cytometry.

Supplementary Table 4 Gene sets used for RT–qPCR.

Supplementary Table 5 Antibodies used for tissue immunostaining.

Supplementary Video 1 Intravital imaging of the mammary fat pad of DPE-GFP:*Rag1*^−/−^ mice highlighting a sessile perivascular macrophage with actively scanning dendrites.

Supplementary Video 2 Intravital imaging of mammary tumors in DPE-GFP:*Rag1*^−/−^ mice showing the localization of sessile perivascular macrophages within the tumor microenvironment.

## Source data

Source Data Fig. 1 Statistical source data.

Source Data Fig. 2 Statistical source data.

Source Data Fig. 3 Statistical source data.

Source Data Fig. 4 Statistical source data.

Source Data Extended Data Fig. 6 Statistical source data.

Source Data Extended Data Fig. 7 Statistical source data.

Source Data Extended Data Fig. 8 Statistical source data.

## Data Availability

Data generated in this study are available at GSE345108, GSE345262 and GSE345263. Source data are provided with this paper.

## Code availability

No new code was used in this study.
