# Supplementary material for: A CD4-lineage erythro–myeloid progenitor within the adult bone marrow defines an alternative pathway for macrophage generation
Source: Nat Immunol. 2026 Oct 26;27(11):2293–305. doi: 10.1038/s41590-026-02686-1 (PMC13710204; doi:10.1038/s41590-026-02686-1)
Supplement: Supplementary file 1 [file 41590_2026_2686_MOESM1_ESM.pdf]

nature immunology

Supplementary information

https://doi.org/10.1038/s41590-026-02686-1

A CD4-lineage erythro–myeloid progenitor within the adult bone marrow defines an alternative pathway for macrophage generation

In the format provided by the authors and unedited

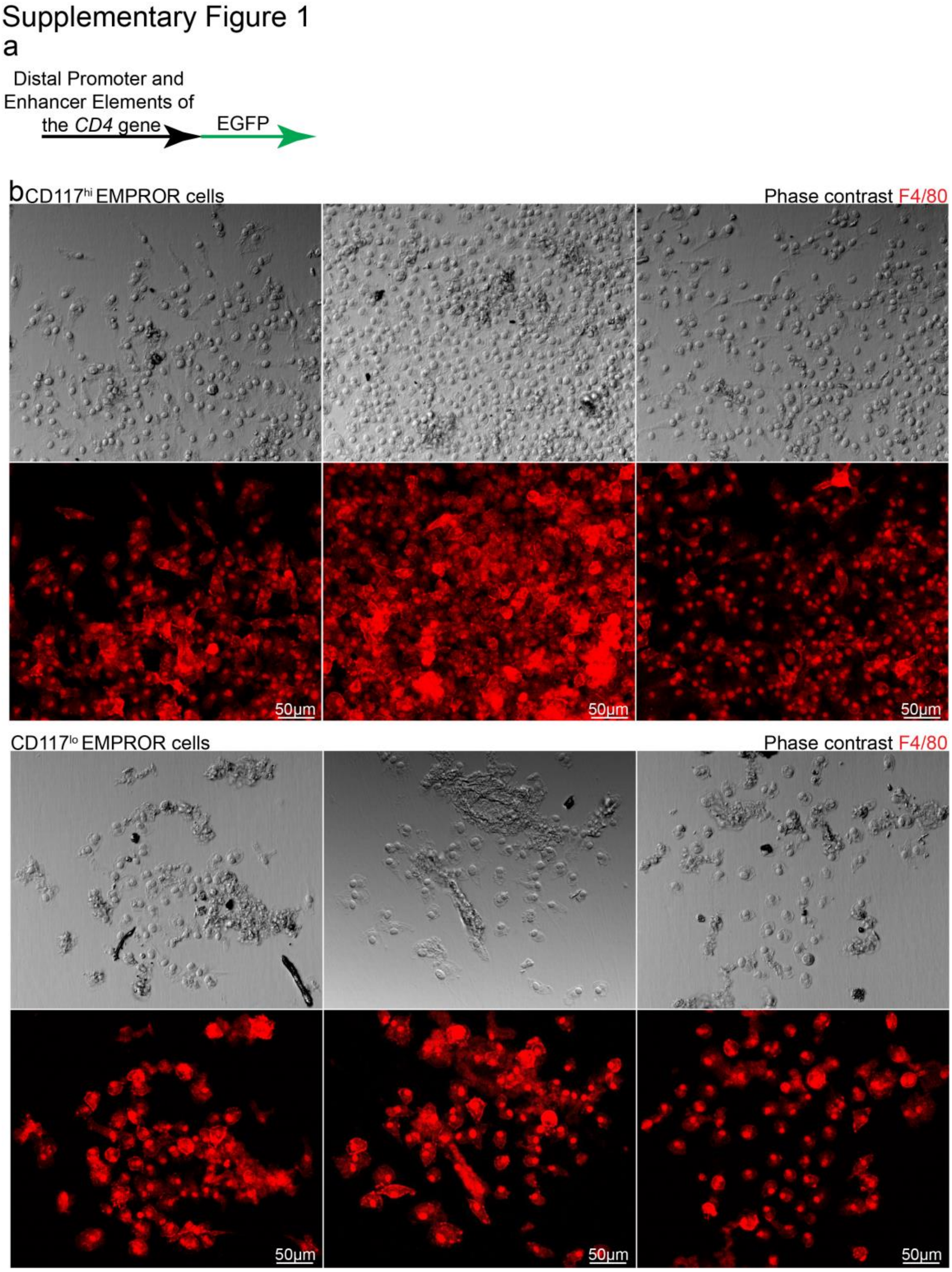

Supplementary Figure 1: Macrophage differentiation potential of CD117^{hi} vs CD117^{lo} EMPROR cells. a, Schematic representation of the construct used to generate the DPE-GFP mice**; b,** Confocal imaging of macrophages generated by in vitro culturing of sorted CD117^{hi} and CD117^{lo} EMPROR cells demonstrating expansion and differentiation into macrophages. Three fields of view are shown for each sample.

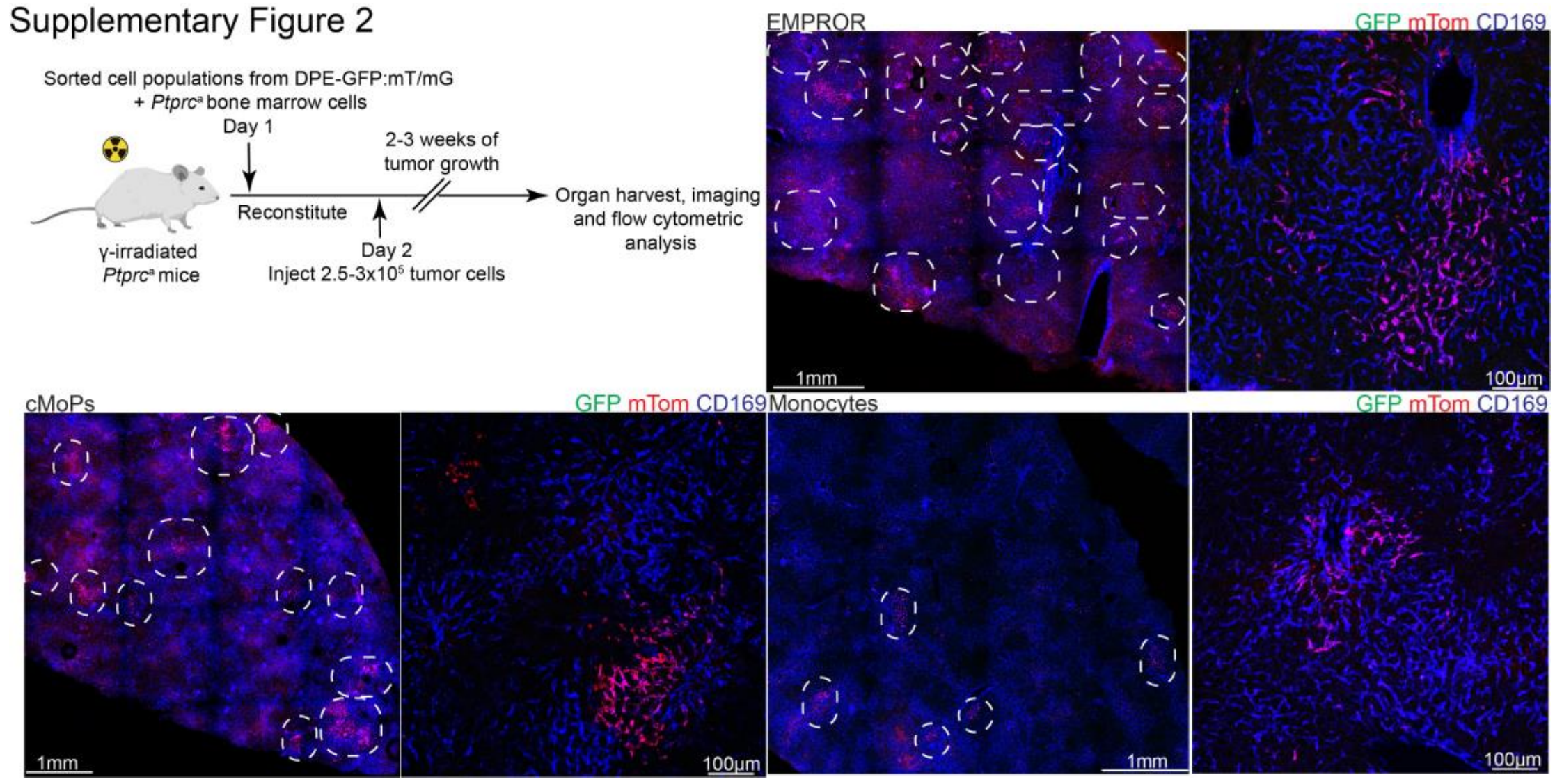

Supplementary Figure 2: Macrophage potential of EMPROR cells, cMoP, and Monocytes. Confocal imaging of immunostained liver sections obtained from mice reconstituted with either EMPROR cells, cMoPs, or monocytes highlights the presence of mTom$^+$CD169$^+$ cells (large field of view). Images are representative of one of three (EMPROR) and one of two (cMoPs and monocytes) independent sorts, adoptive transfer, and tumor implantation experiments.

Supplementary Table 3: Flow Cytometry Antibodies

Antigen	Clone(s)	Fluorophores	Dilution	Cat. Nr.	Vendor
B220	RA3-6B2	BV650	1:200	103241	BioLegend
		PerCP		553093	BD Biosciences
		APC	1:100	553092	
CCR2	475301	PE	1:200	FAB5538P	R&D Systems
CD3e	145-2C11	BV650		564378	BD Biosciences
		PerCP/Cy5.5		551163	
		PE/Cy7		552774	
		APC	1:100	553066	
CD4	RM4-5	PerCP/Cy5.5	1:200	550954	
		BV421		740007	
		APC	1:100	553051	
		PE	1:200	561837	
CD8	53-6.7	APC/Cy7		557654	BD Biosciences
		APC	1:100	553035	
CD11b	M1/70	BV421	1:200	101235	BioLegend
		BV650		101239	BioLegend
		APC/Cy7		557657	BD Biosciences
		FITC		553310	
CD11c	HL3	PE		553802	
		PE/Cy7		558079	
CD19	1D3	BV421		562701	
		PerCP/Cy5.5		551001	
		APC/Cy7	1:100	557655	
CD24	M1/69	PE	1:200	12-0242-82	Thermo Fisher Scientific
CD25	PC61	PE		553866	BD Biosciences
CD34	MEC14.7	BV421	1:20	119321	BioLegend
CD45	30-F11	PerCP/Cy5.5	1:200	550994	BD Biosciences
		APC/Cy7		557659	
CD45.1	A20	PerCP/Cy5.5		45-0453-82	Thermo
CD45.2	104	V500		562129	BD Biosciences
		PerCP/Cy5.5		552950	
		DyLight800 (self-conjugated)		Not applicable	Not applicable
CD64	X54-5/7.1.1	BV421		139309	BioLegend
		PerCP/Cy5.5		139308	
		PE		558455	BD Biosciences
		AF647		558539	
CD71	RI7217	PE/Cy7		113812	BioLegend
CD90.2	53-2.1	PacBlue		140306	
		V500		561616	BD Biosciences
		PE		553005	

CD115	AFS98	PerCP-eFluor 710	1:200	46-1152-82	Thermo Fisher Scientific
		APC		135510	BioLegend
CD117	2B8	PE		553355	BD Biosciences
		PE/Cy7		558163	
CD127	SB/199 (A7R34)	APC	1:100	17-1271-82	Thermo Fisher Scientific
CD150	9D1	APC	1:20	17-1501-81	
CD206	C068C2	PE	1:200	141706	BioLegend
CD209b	ER-TR9	PE		sc-73642	Santa Cruz Biotechnology
CX3CR1	149006 (SA011F11)	PE		149006	BioLegend
CD135 Biotin	A2F10	Not applicable		13-1351-85	Thermo Fisher Scientific
FcγR II/III (CD16/32)	93	PE Cy7	1:80	25-0161-82	Thermo Fisher Scientific
Fc Block	2.4G2	Not applicable	1:1000	553142	BD Biosciences
F4/80	BM8	PerCP/Cy5.5	1:200	123128	BioLegend
		PE		12-4801-82	Thermo Fisher Scientific
		PE/Cy7		123114	BioLegend
Gr-1	RB6-8C5	PE		553128	BD Biosciences
		APC/Cy7	1:100	557661	
IgM	R6-60.2	PerCP/Cy5.5	1:200	550881	
	Clone II/41	APC	1:100	550676	
Ly6C	HK1.4	BV421	1:200	128032	BioLegend
		PE		128008	
Ly6G	1A8	PacBlue		127612	
		PerCP/Cy5.5		560602	BD Biosciences
MHC II	M5/114.15.2	V500		562366	
		PE		107608	BioLegend
		APC-eFluor780		47-5321-82	Thermo Fisher Scientific
NK1.1	PK136	PerCP/Cy5.5		551114	BD Biosciences
		APC		550627	
Sca-1	D7	PerCP/Cy5.5	1:80	45-5981-82	Thermo Fisher Scientific
		PE		12-5981-82	
		Pacific Blue	1:40	108120	BioLegend
Siglec H	551	PerCP/Cy5.5	1:200	129614	
		AF647		129608	
Streptavidin APCCy7	Not applicable	APC/Cy7		554063	BD Biosciences
Streptavidin PECy7	Not applicable	PE/Cy7		557598	
Streptavidin PacBlue	Not applicable	Pacific Blue (PacBlue)		S11222	Thermo Fisher Scientific

Ter119	TER-119	PE		81212-60-25	BioGems
		APC		116212	BioLegend

Supplementary Table 4: RT-PCR gene sets

Genes	Catalogue number	Assay ID	Commercial vendor
Ccr-2	4331182	Mm04207877_m1	Thermo Fischer Scientific
Cd-4	4351372	Mm01185100_m1	
Cx3cr1	4331182	Mm02620111_s1	
Actin	4331182	Mm01304398_m1	
Gapdh	4331182	Mm99999915_g1	

Supplementary Table 5: Immunostaining Antibodies

**Antibody**	**Clone name/Isotype**	**Dilution**	**Vendor**	**Cat. Nr.**
Anti-CD31	MEC 13.3 /Rat IgG 2a	1:200 to 1:400	BD Biosciences	553370
Anti-Laminin	Not applicable/ Rabbit polyclonal IgG	1:400	Sigma Aldrich	L9393-2ML
Anti-F4/80	Rat hybridoma[42]	1:3	Not applicable	
Anti-Iba1	Not applicable/ Rabbit	1:200	Fujifilm Wako Pure Chemical	019-19741

	polyclonal IgG			
Anti-CD163	Not applicable/ Rabbit polyclonal IgG	1:200	Santa Cruz Biotechnology	sc-33560
Anti-CD169	3D6.112/ Rat IgG 2a	1:400	AbD Serotec (now BioRad)	MCA884
Goat anti-Rat IgG Alexa Fluor 647	Not applicable /Goat polyclonal IgG	1:750	Thermo Fisher Scientific	A21247
Goat anti-Rat IgG Alexa Fluor 594				A11007
Goat anti-Rabbit IgG Alexa Fluor 647				A21245
Goat anti-Rabbit IgG				A48254

Alexa Fluor 405				
Goat anti-Rat IgG Alexa Fluor 405		1:100		A48261
Donkey anti-Rat IgG Alexa Fluor 647	Not applicable /Donkey polyclonal IgG	1:100	Abcam	Ab150155
