# Supplementary material for: A CD4-lineage erythro–myeloid progenitor within the adult bone marrow defines an alternative pathway for macrophage generation
Source: Nat Immunol. 2026 Oct 26;27(11):2293–305. doi: 10.1038/s41590-026-02686-1 (PMC13710204; doi:10.1038/s41590-026-02686-1)
Supplement: Supplementary file 3 [file 41590_2026_2686_MOESM5_ESM.pdf]

Supplementary Table 3: Flow Cytometry Antibodies

Antigen	Clone(s)	Fluorophores	Dilution	Cat. Nr.	Vendor
B220	RA3-6B2	BV650	1:200	103241	BioLegend
		PerCP		553093	BD Biosciences
		APC	1:100	553092	
CCR2	475301	PE	1:200	FAB5538P	R&D Systems
CD3e	145-2C11	BV650		564378	BD Biosciences
		PerCP/Cy5.5		551163	
		PE/Cy7		552774	
		APC	1:100	553066	
CD4	RM4-5	PerCP/Cy5.5	1:200	550954	
		BV421		740007	
		APC	1:100	553051	
		PE	1:200	561837	
CD8	53-6.7	APC/Cy7		557654	BD Biosciences
		APC	1:100	553035	
CD11b	M1/70	BV421	1:200	101235	BioLegend
		BV650		101239	BioLegend
		APC/Cy7		557657	BD Biosciences
		FITC		553310	
CD11c	HL3	PE		553802	
		PE/Cy7		558079	
CD19	1D3	BV421		562701	
		PerCP/Cy5.5		551001	
		APC/Cy7	1:100	557655	
CD24	M1/69	PE	1:200	12-0242-82	Thermo Fisher Scientific
CD25	PC61	PE		553866	BD Biosciences
CD34	MEC14.7	BV421	1:20	119321	BioLegend
CD45	30-F11	PerCP/Cy5.5	1:200	550994	BD Biosciences
		APC/Cy7		557659	
CD45.1	A20	PerCP/Cy5.5		45-0453-82	Thermo
CD45.2	104	V500		562129	BD Biosciences
		PerCP/Cy5.5		552950	
		DyLight800 (self-conjugated)		Not applicable	Not applicable
CD64	X54-5/7.1.1	BV421		139309	BioLegend
		PE		558455	BD Biosciences
		AF647		558539	
CD71	RI7217	PE/Cy7		113812	BioLegend
CD90.2	53-2.1	PacBlue		140306	
		V500		561616	BD Biosciences
		PE		553066	
CD115	AFS98	PerCP/Cy5.5	1:200	46-1152-82	Thermo Fisher Scientific
		APC		135510	BioLegend

CD117	2B8	PE		553355	BD Biosciences
		PE/Cy7		558163	
CD127	SB/199 (A7R34)	APC	1:100	17-1271-82	Thermo Fisher Scientific
CD150	9D1	APC	1:20	17-1501-81	
CD206	C068C2	PE	1:200	141706	BioLegend
CD209b	ER-TR9	PE		sc-73642	Santa Cruz Biotechnology
CX3CR1	149006 (SA011F11)	PE		149006	BioLegend
CD135 Biotin	A2F10	Not applicable		13-1351-85	Thermo Fisher Scientific
FcγR II/III (CD16/32)	93	PE Cy7	1:80	25-0161-83	Thermo Fisher Scientific
Fc Block	2.4G2	Not applicable	1:1000	553142	BD Biosciences
F4/80	BM8	PerCP/Cy5.5	1:200	123128	BioLegend
		PE		12-4801-82	Thermo Fisher Scientific
		PE/Cy7		123114	BioLegend
Gr-1	RB6-8C5	PE		553128	BD Biosciences
		APC/Cy7	1:100	557661	
IgM	R6-60.2	PerCP/Cy5.5	1:200	550881	
	Clone II/41	APC	1:100	550676	
Ly6C	HK1.4	BV421	1:200	562727	
		PE	1:200	128008	BioLegend
Ly6G	1A8	PacBlue		127612	
		PerCP/Cy5.5		560602	BD Biosciences
MHC II	M5/114.15.2	V500		562366	
		PE		107608	BioLegend
		eF780		47-5321-82	Thermo Fisher Scientific
NK1.1	PK136	PerCP/Cy5.5		551114	BD Biosciences
		APC		550627	
Sca-1	D7	PerCP/Cy5.5	1:80	45-5981-82	Thermo Fisher Scientific
		PE		12-5981-82	
		Pacific Blue	1:40	108120	BioLegend
Siglec H	551	PerCP/Cy5.5	1:200	129614	
		AF647		129608	
Streptavidin APCCy7	Not applicable	APC/Cy7		554063	BD Biosciences
Streptavidin PECy7	Not applicable	PE/Cy7		557598	
Streptavidin PacBlue	Not applicable	Pacific Blue (PacBlue)		S11222	Thermo Fisher Scientific
Ter119	TER-119	PE		81212-60-25	BioGems
		APC		116212	BioLegend
