# Supplementary material for: A CD4-lineage erythro–myeloid progenitor within the adult bone marrow defines an alternative pathway for macrophage generation
Source: Nat Immunol. 2026 Oct 26;27(11):2293–305. doi: 10.1038/s41590-026-02686-1 (PMC13710204; doi:10.1038/s41590-026-02686-1)
Supplement: Supplementary file 4 [file 41590_2026_2686_MOESM6_ESM.pdf]

Supplementary Table 4: RT-PCR gene sets

Genes	Catalogue number	Assay ID	Commercial vendor
Ccr-2	4331182	Mm04207877_m1	ThermoFischer Scientific
Cd-4	4351372	Mm01185100_m1	
Cx3cr1	4331182	Mm02620111_s1	
Actin	4331182	Mm01304398_m1	
Gapdh	4331182	Mm99999915_g1	
