# Supplementary material for: A CD4-lineage erythro–myeloid progenitor within the adult bone marrow defines an alternative pathway for macrophage generation
Source: Nat Immunol. 2026 Oct 26;27(11):2293–305. doi: 10.1038/s41590-026-02686-1 (PMC13710204; doi:10.1038/s41590-026-02686-1)
Supplement: Supplementary file 5 [file 41590_2026_2686_MOESM7_ESM.pdf]

Supplementary Table 5: Immunostaining Antibodies

Antibody	Clone name/Isotype	Dilution	Vendor	Cat. Nr.
Anti-CD31	MEC 13.3 /Rat IgG 2a	1:200 to 1:400	BD Biosciences	553370
Anti-Laminin	Not applicable/ Rabbit polyclonal IgG	1:400	Sigma Aldrich	L9393-2ML
Anti-F4/80	Rat hybridoma[42]	1:3	Not applicable	
Anti-IbaI	Not applicable/ Rabbit polyclonal IgG	1:200	Fujifilm Wako Pure Chemical	019-19741
Anti-CD163	Not applicable/ Rabbit polyclonal IgG	1:200	Santa Cruz Biotechnology	sc-33560
Anti-CD169	3D6.112/ Rat IgG 2a	1:400	AbD Serotec (now BioRad)	MCA884
Goat anti-Rat IgG Alexa Fluor 647	Not applicable /Goat polyclonal IgG	1:750	Thermo Fisher Scientific	A21247
Goat anti-Rat IgG Alexa Fluor 594				A11007
Goat anti-Rabbit IgG Alexa Fluor 647				A21245

Goat anti-Rabbit IgG Alexa Fluor 405				A48254
Goat anti-Rat IgG Alexa Fluor 405		1:100		A48261
Donkey anti-Rat IgG Alexa Fluor 647	Not applicable /Donkey polyclonal IgG	1:100	abcam	Ab150155
